# Total plasma N-glycomic patterns of COVID-19 disease

**DOI:** 10.1007/s10719-025-10201-1

**Published:** 2026-01-20

**Authors:** Georgia Elgood-Hunt, Jack Cheeseman, Richard A. Gardner, Thomas Sénard, Paulina A. Urbanowicz, Alejandro A. Garcia Leon, Cormac McCarthy, Marco P. Monopoli, Daryl L. Fernandes, Craig P. Thompson, Oleg A. Mayboroda, Patrick. W.G. Mallon, Daniel I. R. Spencer

**Affiliations:** 1 Ludger Ltd., Research and Development, Oxford, UK; 2 Division of Biomedical Sciences, Warwick Medical School, University of Warwick, Coventry, UK; 3 Center for Proteomics and Metabolomics, Leiden University Medical Center, Leiden, 2333ZA The Netherlands; 4 Division of Endocrinology and Diabetes Prevention and Care, IRCCS Azienda Ospedaliero-Universitaria di Bologna, Bologna, Italy; 5 Centre for Experimental Pathogen Host Research (CEPHR), School of Medicine, University College Dublin (UCD), Belfield, Dublin 4, Ireland; 6 Department of Respiratory Medicine, St Vincent’s University Hospital, Dublin 4, Ireland; 7 School of Medicine, University College Dublin, Belfield, Dublin 4, Ireland; 8 Department of Chemistry, Royal College of Surgeons in Ireland, 123 St Stephens Green, Dublin, Ireland; 9 Department of Infectious Diseases, St. Vincent’s University Hospital, Dublin 4, Ireland

**Keywords:** COVID-19, Glycans, ICU, Mortality

## Abstract

Total plasma *N*-glycans alter in disease states, with few studies focused on COVID-19. A discovery cohort of 310 COVID-19 patients, replicated with 97 COVID-19 patients and tested with 100 COVID-19 patients, was used to unearth the *N*-glycans capable of distinguishing infection, as well as prognosis of intensive care unit (ICU) admission and mortality. All significant bisected glycans were decreased in patients compared to controls, whilst fucosylated tri-antennary glycans and sialylated tetra-antennary glycans were increased in patients. For both those admitted to the ICU and those who died, Peak 61 (A4G4S4F) was elevated in the more severe disease course, while Peak 29 (FA2G2S2) was lowered. Pinpointing specific glycosylation changes has alluded to a potential story of glycoprotein pathways in response to SARS-CoV-2 infection. This study could be further explored through deriving the glycoproteins associated with the glycans of interest and the glycosylation changes experienced on these proteins.

**Graphical Abstract:**

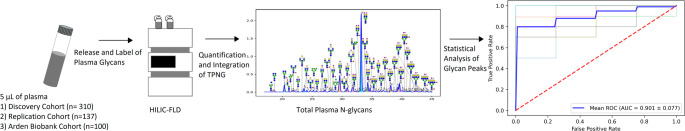

**Supplementary Information:**

The online version contains supplementary material available at 10.1007/s10719-025-10201-1.

## Introduction

Since the initial detection of SARS-CoV-2 in December 2019[1], the virus and the corresponding disease, coronavirus disease 2019 (COVID-19), has had a global impact causing over 7 million deaths (to date, 30 March 2025) [2]. Although millions of individuals have been infected, the symptom profile experienced varies and disease severity can range from asymptomatic, to mild involving fever and cough[3], to severe disease including pneumonia, organ failure and death [4]. The prediction of the infection outcome has been particularly important during the outbreak in order to understand the likelihood of a patient needing ventilation support and hospitalization. The extensive heterogeneity of the disease places emphasis on early-infection predictors of disease outcome, to allow for appropriate treatment plans and minimize mortality. Although COVID-19 incidences have fallen, there is still a persistent presence of drifted strains which have become endemic. Consequently, it is important to identify early predictors of disease severity. Here, we analyse these features in samples collected in 2020–2021, prior to vaccine distribution.

Risk factors and associations of COVID-19 severity have been investigated in several studies, utilising a variety of patient information, including comorbidities such as the higher incidence of hypertension, heart failure, diabetes mellitus and coronary heart disease in COVID-19 severe cases [5]. Clinical markers such as C-reactive protein (CRP), Interleukin 6 (IL-6), and erythrocyte sedimentation rate (ESR) were found to be increased in severe COVID-19 cases [6]. Mortality prediction has been built by age, O2 saturation, hydroxychloroquine during treatment, maximum body temperature and patient interaction (e.g. outpatient, inpatient, telehealth), achieving a 0.91 AUC [7].

In addition to this, pathological findings have highlighted that disruption in the alveolar epithelium drives the consequential escalation of disease [8]. An unregulated inflammatory response stimulated by SARS-CoV-2 causes additional damage, for instance a low platelet count is associated with severe disease [9]which can be prevented with prophylactic anticoagulant treatment [10] and changes in coagulation parameters as indicated by d-dimer increase [11] However, due to the pathophysiology of SARS-CoV-2 which encompasses these severity risk factors not being fully characterised, the personalisation of the treatments is limited. Although genomics and proteomics have been used to show insight into the cellular mechanism of SARS-CoV-2 infection, additional understanding of protein interactions is seen through post-translational modifications, particularly glycosylation. *N*-glycans are capable of influencing cell signaling, immune modulation [12], reflected with glycosylation changes driven by immune responses [13]. For instance, SARS-CoV-2 projects glycosylated spike glycoproteins on the viral surface to enable host cell entry [14]. Host cell viral susceptibility is also impacted by glycans, such as heparan sulfate, a glycosaminoglycan, present on host cells and extracellular matrix, which acts as an assistant receptor for SARS-CoV-2 entry [15]. Whilst glycans also affect the immune response, with immunoglobulin G (IgG) presenting distinct glycosylation profiles in COVID-19 individuals[16, 17]. For example, inflammatory glycosylation modifications, e.g. fucosylated agalactosylated IgG, were increased in cases of hospitalization when compared to milder forms. In addition, mild patients experience a rise in plasma IgG sialic acids over time, which could be explained by higher levels of sialic acid on the IgG CH2 domain acts as a protection against inflammation activation and potentially prolongs the lifespan of anti-SARS-CoV-2 antibodies, driving anti-inflammatory responses [18].

Plasma glycans are useful disease biomarkers, the importance of which has been showcased in diabetes [19], systemic lupus erythematosus [20], colorectal cancer [21], cardiovascular disease [22] and inflammatory bowel disease [23]. It is therefore evident that total plasma *N*-glycans (TPNG) can imply the disease state of an individual and expanding on the information gained from IgG, looking outside of the humoral immune response, and encompassing the innate and cellular immune response. This is particularly important in the context of COVID-19 as SARS-CoV-2 infection triggers a broad immune response, such as a hyperactivated immune response from viral RNA sensing, dysregulated neutrophil extracellular traps (NET) leading to thrombosis and syncytia formation [24]. By investigating the relationship of TPNG with COVID-19 disease, it can add to the evidence of the utility of TPNG in disease biomarkers and serves as an example of methodology for other diseases.

Recent developments in plasma and serum sample quantification, such as Hydrophilic Interaction Liquid Chromatography with florescence detection (HILIC-FLD), have rendered TPNG a viable source of biomarkers and clinical application, due to the development of automation and high-throughput techniques. However, few studies have investigated the predictive capacity of plasma *N*-glycan for COVID-19 disease outcomes. A cohort of 196 COVID-19 patients has shown promise in distinguishing mild from critical disease by the glycan, Fuc1Hex5HexNAc5 (AUC = 0.88; *p* < 0.001), potentially FA3G2, in addition a decrease in fucosylation has been established in COVID-19 patients [25]which has been linked to an upregulation of antibody-dependent cell cytotoxicity in the acute immune response [26]. Other studies have utilised MALDI-MS to uncover specific glycan structures capable of distinguishing mild and severe COVID-19 patients. Based on 169 COVID-19 patients and 12 healthy controls, Bladergroen et al., found glycans such as overall sialylation of tetra-antennary glycans (TA4S), linked to liver damage, and A4G4S4/H7N6E4, both strongly correlated with disease severity and H7N6E4 was raised in ICU admission individuals [27]. In addition, COVID-19 infected individuals could be distinguished from healthy controls, mainly by sialylated glycans, typically linked to acute-phase proteins [28], for example, highly sialylated tetra-antennary *N*-glycans are linked to alpha-1-acid glycoprotein (A1AGP) [29].

Multiple studies have found that individual serum or plasma glycans have the strength to associate with COVID-19 disease diagnosis and prognosis, which could further be built on with the inclusion of multiple glycans and clinical information in multivariate modelling[25, 27, 30] These studies highlight that analysis of glycans can inform us of the broader disease environment through proposed protein links. However, whilst these studies are informative, there are still likely to be several glycoprotein candidates that would require further investigation. In addition, greater samples sizes, particularly when considering controls, and an external replication cohort could expand on such results, to demonstrate the reproducibility of glycans as a biomarker of disease.

We explored TPNG as biomarkers for COVID-19 disease and its prognosis, answering the demand for a larger control cohort and replication of significant glycomic biomarkers with an external cohort. Patients infected from the first wave of COVID-19 during March 2020 to September 2021, as part of the AIID cohort (All Ireland Infectious Diseases), had plasma samples collected upon symptom onset. To expand the known associations of glycan composition with SARS-CoV-2 infection, the plasma *N*-glycome was quantified via ultra-high performance liquid chromatography (UHPLC-FLD) with fluorescence detection and assessed for biomarker potential for disease severity, ICU admission and mortality likelihood. The AIID cohort serves as the discovery and replication cohort used in the analysis for univariate and multivariate modelling. Glycans that showed statistical significance were tested with an independent cohort collected by the Arden Biobank at University Hospitals Coventry and Warwickshire (UHCW), referred to as the Arden Biobank cohort. The results highlight the capabilities of glycans as a marker of infection and a predictor of severe disease course. This method applied to other disease, could prove valuable in raising attention to patients who are at risk of ICU admission or escalation to mortality early in the disease course and provide opportunity for preventative treatment.

## Method

### Sample acquisition and processing

Plasma samples were acquired from 2 cohort studies, namely, All Ireland Infectious Diseases (AIID), which is a multicenter cohort of individuals relating to Infectious Disease in Ireland, and the Warwick Arden Biobank cohort, focusing on individuals infected with SARS-CoV-2 and admitted to the intensive care unit (ICU).

AIID participants were selected by a confirmed COVID-19 infection between March, 2020 and September 2021. Plasma samples were collected upon symptom onset. Individuals were divided by disease severity, including mild, moderate, severe and critical, as defined by the World Health Organisation (WHO) [31].

**Fig. 1:**
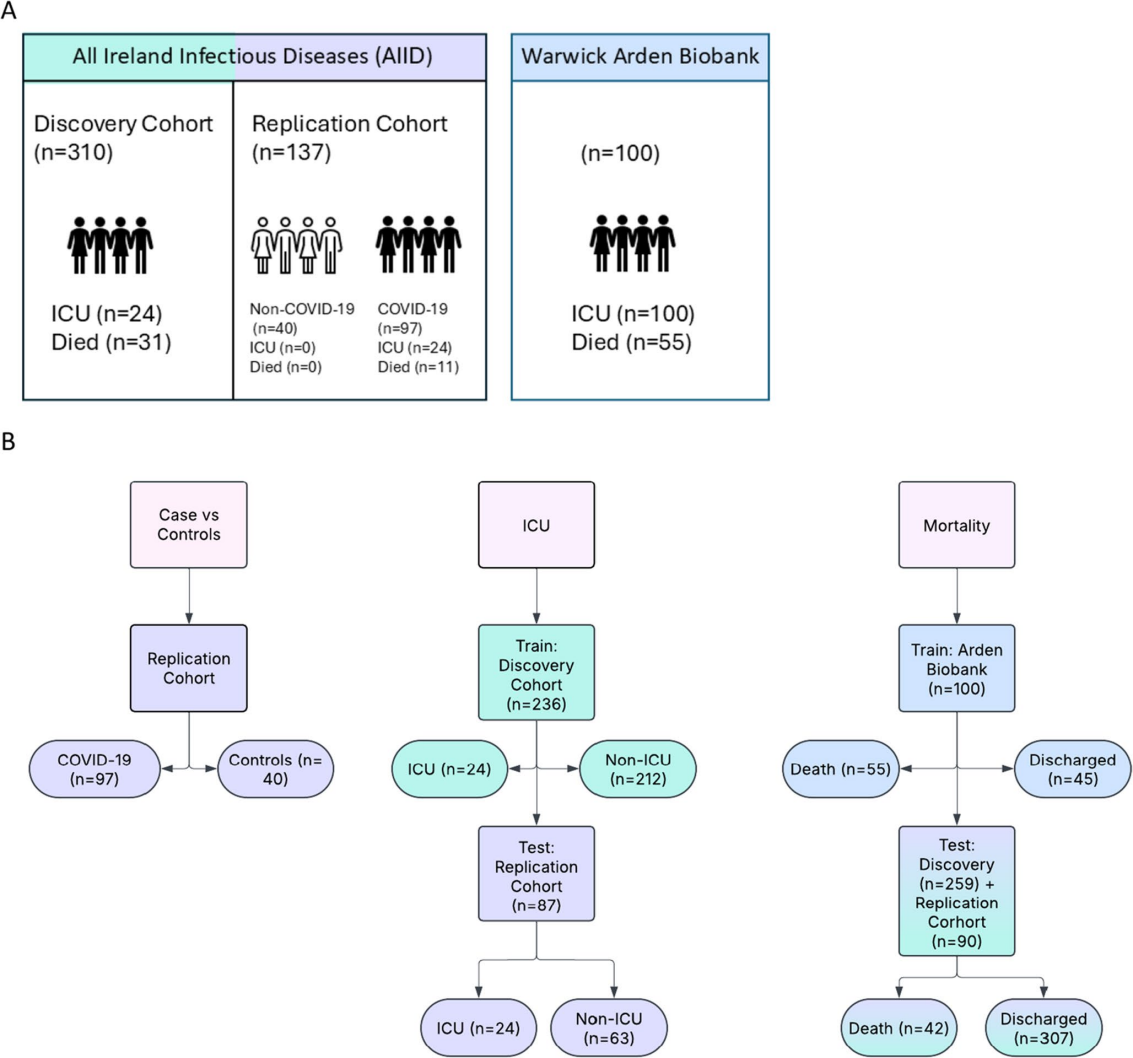
Infographic depicting the utilization of the discovery, replication and Arden Biobank cohorts. **A**. Table displays the number of patients within the discovery and replication cohort, derived from the same origins, and the number of known patients experiencing ICU admission and mortality. In addition, the number of patients in the Arden Biobank. **B**. A flowchart of how a cohort was used for the 3 design questions, including COVID-19 disease compared to controls, ICU admission and mortality. Numbers may vary across cohorts depending on design questions due to removal of patients with missing data, as demonstrated in figure 1B. Figure 1A total cohort number includes patients with missing information required for analysis in figure 1b

The AIID cohort included 2 sub-cohorts, processed separately at different times, referred to as discovery and replication. The discovery cohort is composed of 310 patients containing 151 mild, 66 moderate, 42 severe and 51 critical COVID-19 disease defined by WHO, 212 remained out of the ICU, whilst 24 were admitted, 228 were discharged from hospital and 31 died, as shown in Fig. 1 (Document S3 Table 1). The Replication cohort is composed of 97 COVID-19 patients, containing 40 mild, 22 moderate,15 severe and 15 critical COVID-19 disease, with 24 admitted to ICU and 63 remained out of the ICU, 79 patients were discharged from hospital and 11 died (Document S3 Table 2). In addition, the replication cohort contains 40 non-COVID-19 patients, exclusively analysed during the comparison of COVID-19 patients and controls and was not included in severity, ICU, and mortality analysis, as shown in Fig. 1. All non-COVID-19 patients did not enter ICU or experience mortality during the study (Document S3 Table 4). The AIID cohort consisted of UHPLC-FLD-derived glycomic data, general patient information including comorbidities, in addition, clinical information. Cohort numbers used in different analysis e.g. severity, ICU, mortality, case vs. controls, may vary depending on missing critical information needed to perform the design question, as those patients will be excluded.

The cohort provided by Arden Biobank was utilised to build and train the model for mortality outcome given its complete independence from the AIID cohort and its balanced nature. This cohort consists of 100 patients, all of which have been admitted to the ICU, 45 discharged from hospital whilst 55 died, as shown in Fig. 1(Document S3 Table 3). WHO-classified disease severity information was not available. In addition to the UHPLC-FLD-derived glycomic data, comorbidities and general patient information were provided; however clinical information wasn’t available.

### Sample processing

To each well of a skirted 96-well plate (4titide Ltd, 180 µL volume) 5 µL of either patient plasma sample, plasma standard or IgG standard was added. The plasma and IgG standards were run in triplicate, randomly distributed across the plate. The volume was made up to 9 µL by addition of 4 µL water. Followed by 1 µL of denaturation buffer (NEB). The samples were vortexed and briefly centrifuged before incubation for 10 min at 100 °C. The plate was then transferred to a liquid handling robot (Hamilton, Birmingham, UK) for semi-automatic sample processing. After the samples cooled to room temperature, an additional 5 µL water, 1 µL NP-40 (NEB), 2 µL reaction buffer (NEB) and 2 µL PNGase-F solution (NEB) were added to each sample. The samples were vortexed and briefly centrifuged and then incubated at 37 °C for 16 h. The samples were dried using a vacuum centrifuge (Thermoscientific) for 2 h. The glycans were then converted to aldoses by addition of 20 µL 1% formic acid solution. Again, the samples were vortexed and briefly centrifuged prior to incubation for 45 min at room temperature. To remove excess protein, the samples were then filtered through a LudgerClean 96-well Protein Binding Membrane Plate with water (2 × 100 µL).

The resulting 200 µL solution was transferred to a non-skirted 96-well plate (4titude Ltd, 300 µL volume) and dried down in a vacuum centrifuge for 9 h. The dried glycans were fluorescently labelled by reductive amination with 20 µL procainamide labelling solution (Ludger) [32]. The samples were, again, vortexed and briefly centrifuged prior to incubation for 1 h at 65 °C. The samples were combined with 80 µL acetonitrile before being transferred to a LudgerClean Procainamide Cleanup Plate. The samples were then washed with acetonitrile (3 × 100 µL) to remove excess labelling solution. The labelled glycans were recovered by washing with water (2 × 100 µL).

For each sample, 100 µL were added to 300 µL of acetonitrile, in preparation for UHPLC-FLD-MS/MS analysis. To prepare the system suitability standards (plasma, IgG, glucose homopolymer and blanks 25 µL of each standard was added to 75 µL acetonitrile. Both standards and samples were run through the HILIC-FLD coupled to mass spectrometry (MS). A volume of 20 µL of each standard and sample was injected onto an ACQUITY UPLC BEH-Glycan 1.7 μm,2.1 mm×150 mm column (Waters) at 40 °C on a Thermoscientific Vanquish UHPLC instrument fitted with a fluorescence detector (λex = 310 nm,λem = 370 nm), then sprayed in-line into a Thermoscientific Orbitrap Exploris 120 mass spectrometer without splitting the flow. The chromatography conditions used were: Solvent A 50 mM ammonium formate pH 4.4 made from LudgerSep *N*-Buffer, and solvent B acetonitrile. Gradient conditions were 0 to 53.5 min, 76 to 51% B, 0.4 mL/min; 53.5 to 55.5 min, 51% to 0% B, 0.4 mL/min to 0.2 mL/min; 55.5 to 57.5 min,0% B at a flow rate of 0.2 mL/min; 57.5 to 59.5 min, 0 to 76% B, 0.2mL/min; 59.5 to 65.5 min, 76% B, 0.2 mL/min; 65.5 to 66.5 min,76% B, 0.2 mL/min to 0.4 mL/min; 66.5 to 70.0 min, 76% B, 0.4 mL/min. The Exploris 120 settings were source temperature, 300 °C; gasflow, 10 L/min; capillary voltage, 3500 V; max accu time, 100 ms; positive ion mode; mass range scanned, 500 − 2500.

HappyTools [33] (version 0.1-beta1, build 190115a, start time 10, end time 60) was used to integrate and quantify HILIC fluorescence outputs for each sample, generating a chromatogram composed of 62 Peaks for each plasma sample and plasma standard, as shown in figure S3. Measurements were normalized by relative area, where the area of each peak was divided by the total area of the chromatogram. Outputs from mass spectrometry were processed through ThermoScientific Freestyle software to indicate which glycans were represented in the UHPLC-FLD glycan peaks. MS outputs were processed initially with LacyTools [34] to allow for ease of alignment and then quantified in Skyline [35]. Whilst MS data was not analysed or included in classifier models, it was utilized to confirm glycan structures under UHPLC-FLD peaks (Document S2). In cases where multiple glycans presented under a single peak, the glycan of highest abundance was focused on.

### Clinical data processing

This data was processed and provided by the School of Medicine and Centre for Experimental Pathogen Host Research at the University College Dublin, alongside St Vincent’s University Hospital under the AIID cohort study. The clinical biomarkers analysis was performed on frozen ethylenediaminetetraacetic acid (EDTA)-derived plasma. Prior to analysis, samples were stored at Ultra Low Temperature freezers (−80°). 36 biomarkers were analysed, including several different inflammatory pathways. Simultaneous measurement of circulating biomarkers (Multiplex) was perform using the electrochemiluminescence technology from Meso Scale Discovery (MSD, Rockland, MD, USA) and fluorescence bead-based technology from Luminex^®^ (Luminex^®^, R&D.

Systems, Minneapolis, MN, USA). MSD’s immunoassay kits were used to measure 10 biomarkers (IL 10, IL-12, IL-2, IL-6, IL1 beta, IL-5, IL17A, IL 13, IL-4, TNF alpha/Cat No. K15067L-2), and 4 biomarkers.

(IFN-a2, IFN-b, IFN-g, IFN-l1/Cat No. K15094K-2). R&D Systems/Luminex immunoassay kit was used to measure 22 biomarkers (sCD40L, sP-selectin, D-dimer, ICAM-1, E-selectin, sCD163, MCP1, MIP1-alpha, IL-18, IP10(CXCL10), IL8, IL1RA, PD-L1, IFN-l2 (IL28A), IFN-l3 (IL-28B), VEGF, EGF, PDGF-AA, TPO, S100B, TGF (alpha), GM CSF/Cat No. LXSAHM-22). To avoid freeze thaw effect, single-use aliquots were implemented. Samples were run in duplicate. Controls were featured on every plate. Samples which fell out of 10% intra-plate coefficient of variation (CV) and 15% interpolate CV, were rerun. If the sample still failed to lie within the acceptable CV after 2–3 reruns, they were deemed unreliable and removed from analysis.

### Data preprocessing

A Principal Component Analysis (PCA) was performed to assess the variation within the data and assess whether outliers were present. Samples which failed to run correctly on the UHPLC-FLD and identified by PCA and chromatogram inspection were rerun. Batch effect was tested through PCA across the 3 cohorts to account for any retention time shifts arising from chromatographic variation over the span of the data acquisition period. If required, correction was applied through the analysis of covariance (ANCOVA) framework [36]. In the case of a glycan peak splitting into 2, it is combined to allow ease of comparison across plates, unless peak splitting is consistent across all plates.

### Statistical analysis

#### Univariate analysis

All statistical analysis was performed via Python, version 3.11.7. Clinical markers were normalised within each cohort to ensure comparability. Categorical values were encoded into indicator variables. Missing continuous values were imputed with the mean and categorical variables with the most frequent value, when the proportion of missing values was less than 10%. Glycomic data was standardised to remove the impact of different peak ranges by removing the mean and scaling to unit variance.

As part of the AIID cohort, there was a subset of 40 individuals who were admitted to hospital but were not experiencing inflammation or COVID-19, contained in the replication cohort. Morbidities of controls are summarized in document S3 Table 4. This subset was taken as a control and compared to COVID-19 infected individuals to establish the changes in the glycomic profile caused by COVID-19 infection. The strongest glycan differentiators between these groups were extracted by a Volcano plot with absolute fold change and Mann-Witney U test. For each glycan peak, fold change was calculated with the mean relative area of COVID-19 patients/mean relative area of control patients. Glycan peaks which followed the criteria (|fold-change| > 1.5 and *p*-value < 0.05 after Benjamini-Hochberg Correction) were selected as significantly different across COVID-19 patients and controls.

Pearson correlation between glycans and clinical markers was assessed to determine any underlying relationships. Univariate analysis was applied on the discovery and replication cohort. Kruskal-Wallis Test determined the significance of glycans and clinical markers across WHO Severity. Ordinal Model based on a logistic distribution captured the ascending order from mild to critical disease. In addition, the significance of biomarkers across ICU admission and mortality was tested via Mann-Whitney U test. Chi-squared was used for categorical variables. Benjamini-Hochberg Correction was applied to account for multiple testing. Univariate predictive power was measured by 10-fold cross-validated logistic regression with age and sex correction, taking AUC as the performance metric.

To further narrow down the search for strong glycan biomarkers, an additional selection criteria was applied, taking into account fold change. This being mean relative area of peak values for ICU admitted patient/Non-admitted, for ICU admission analysis. For mortality analysis, the mean relative area of the peak for those who died/those who were discharged. Glycan peaks which followed the criteria (|fold-change| > 1.5 and *p*-value < 0.05 after Benjamini-Hochberg Correction) were selected as the glycans of interest.

Figures of glycan structures were created in GlycoWorkbench (version 2.1)[37], with symbols representing monosaccharides.

#### Multivariate analysis

To explore how the glycomic profile differs across COVID-19 severity and ICU admission, discovery cohort was used to train and validated by the replication cohort to confirm significant results and performance of prediction models. When building the mortality model, the Arden Biobank cohort was used as a training cohort due to its balanced nature, with discovery and replication combined for validation. Clinical data was not available for the Arden Biobank cohort.

Several multivariate classification models were built to predict COVID-19 disease, WHO severity, ICU admission, and mortality outcome. Multiclass classifier models for WHO severity used one-vs-rest comparison of severity group performance, with a weighted-mean average Area Under Curve (AUC) used to provide an overall performance of the model. The classification model used is Gaussian Naive Bayes. Assumptions were tested for adherence. Kernel density estimate plots were used to assess the variable distribution. Variable independence was checked by VIF. Covariates, namely age and sex, were tested for influence on glycan peaks in the model. Models including age and sex were compared to the baseline model using generalized likelihood ratio test. In the case of ICU models, sample weights were included to compensate for the unbalanced nature of the cohorts. Sample weights were calculated by:

Number of samples/(number of classes * number samples in class) based on Logistic Regression in Rare Events Data, King, Zen, 2001.

The model performance was demonstrated over a 10-fold cross validation, using AUC as the performance metric. Glycans, clinical markers and patient information were all considered for the multivariate models, with feature method, sequential feature selection. Feature selection was guided to select models including 3–4 features to prevent multicollinearity, in the cases of long models, collinearity checks were in place to ensure reproducibility. Variable Inflation Factor (VIF) tested the multicollinearity between variables selected in the model and those with a VIF greater than 3 were deselected. C-reactive protein (CRP) was used as a baseline comparison for predicting WHO severity, ICU admission, and mortality. Final models were displayed as a Receiver Operating Characteristics (ROC) curve, with 5-fold cross validation for visual simplicity.

## Results

The *N*-glycan composition from plasma was firstly compared across those with and without COVID-19 to allude to any glycosylation changes driven by the disease. The replication cohort consisted of 97 COVID-19 patients and as a comparison, 40 non-inflamed, non-infected individuals who were still admitted to the hospital. The distribution of these 2 groups were not well distinguished by PCA (figure S5). Glycans raised as significant across COVID-19 patients and controls were cross-referenced with previous studies to determine the reproducibility of the biomarkers [27]. Secondly, an exploration of how N-glycan levels are indicative of COVID-19 disease progression, namely ICU admission and mortality outcome, are performed. This analysis harnesses the power of having 3 cohorts (Discovery, Replication and Arden Biobank) with considerable patient numbers to assess and confirm patterns witnessed.

### N-glycan signature of COVID-19 infection

Univariate analysis with multiple testing correction revealed 32 out of the 62 glycan peaks to be significantly different across COVID-19 patients and controls (Document S4 Tables 1, 2 and 3). To further distinguish strong biomarkers of disease, a stricter selection criterion was applied (|fold-change| > 1.5).

**Fig. 2:**
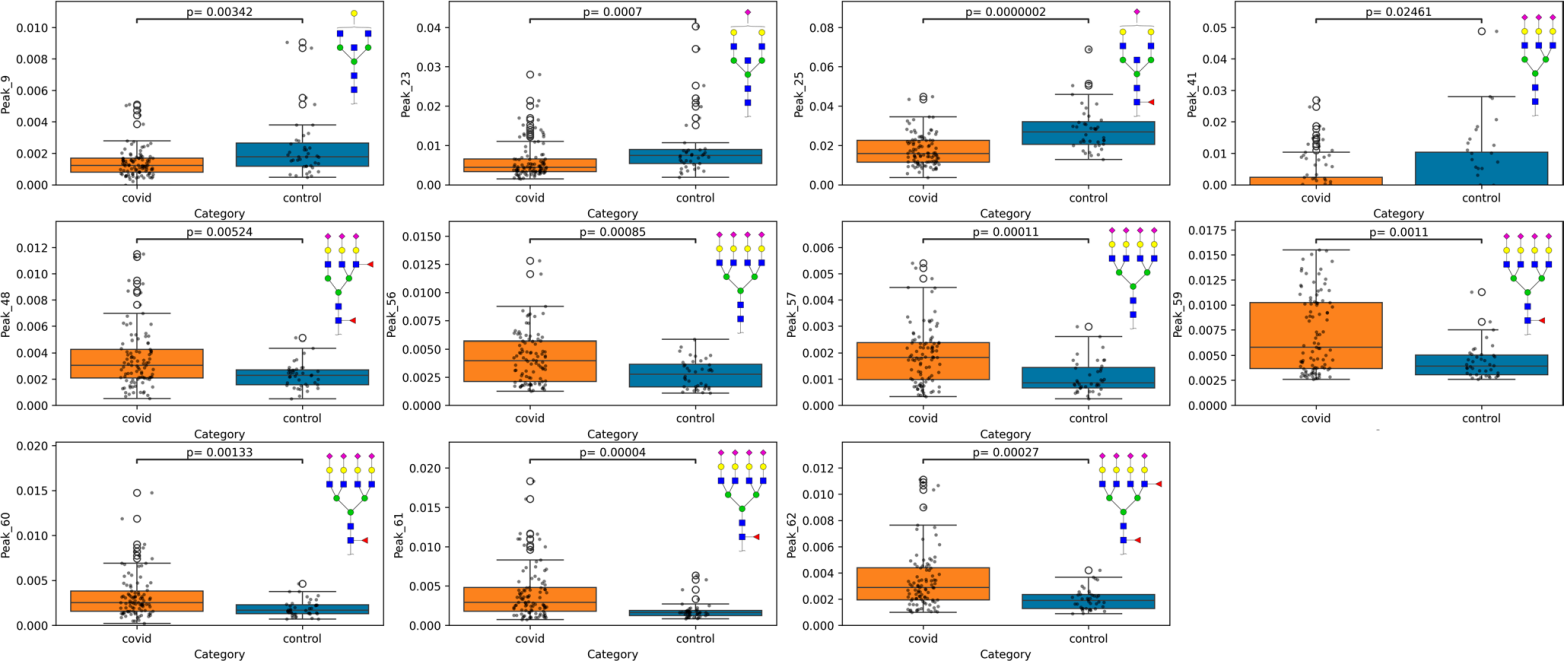
Boxplots depicting the relative area of glycan peaks (y-axis) significantly different across case vs controls (x-axis) with selection criteria (|fold-change| > 1.5 and*p*-value < 0.05 after Benjamini-Hochberg Correction). *P*-values have been produced from the Mann-Witney U test with multiple testing correction applied. Glycan structures provided

As shown in Fig. 2, the boxplots depicting case vs. controls highlighted 11 glycans to be strongly significant. Of the significant glycans, as shown in Fig. 2, all glycans with bisection (Peaks 9,23,25) were decreased in patients compared to controls, whilst fucosylated triantennary glycans (Peak 48) and sialylated tetra-antennary glycans (Peaks 56,57,59,60,61,62) were increased in patients.

**Fig. 3:**
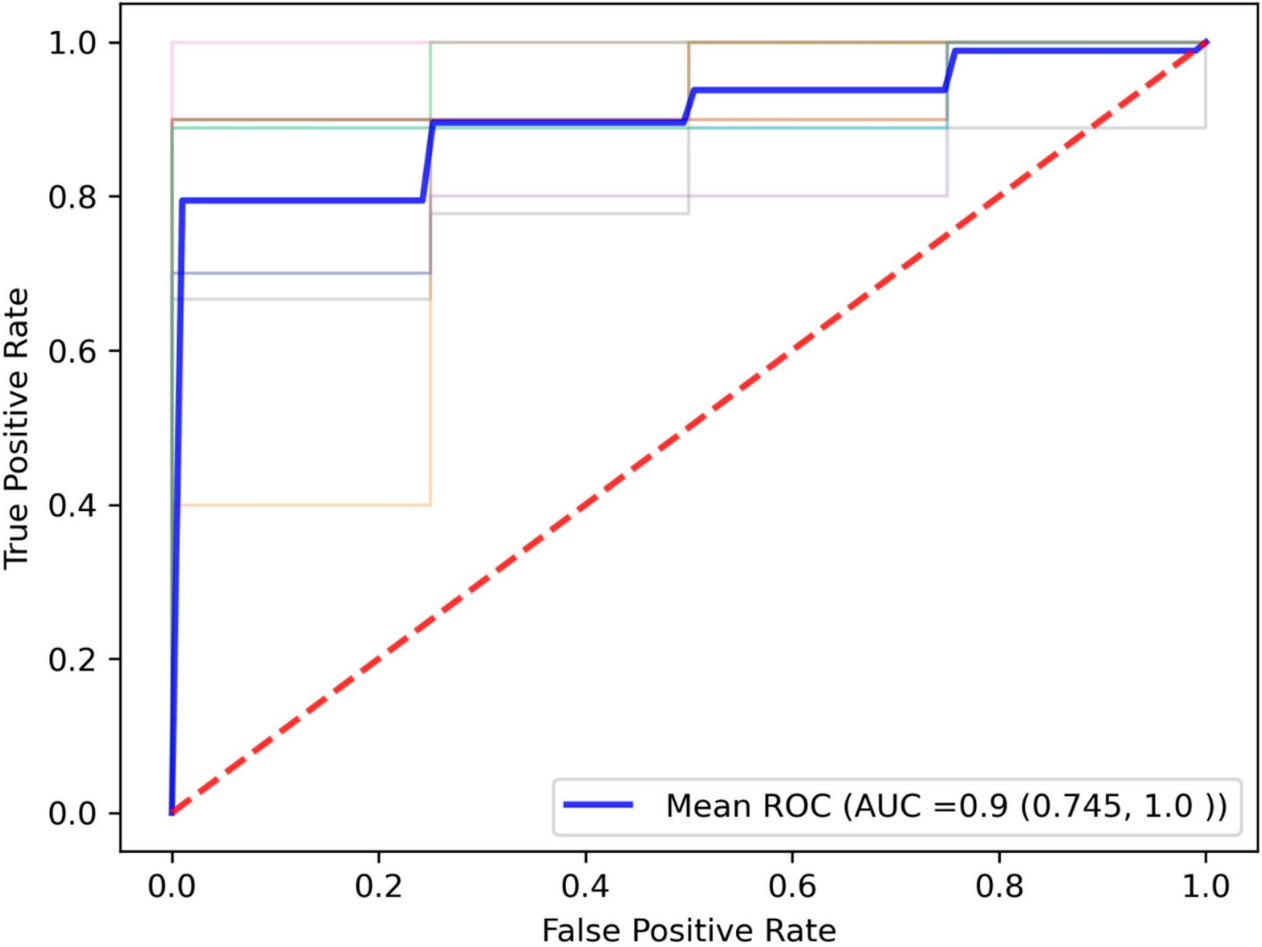
A ROC curve depicting the performance of the Gaussian Naïve Bayes model distinguishing healthy controls vs COVID-19 patients utilising 5-fold cross-validation, which achieves a 0.90 AUC. The dashed red line represents an AUC of 0.5. The ‘Mean ROC’ line in blue represents the average performance of the model across the 5 folds, whilst all other coloured lines are the performance of each fold in the cross-validation. The model is composed of Peaks 22, 33, 56 and 25. With VIF values all below 3

Through sequential feature selection of all glycan peaks, a classification model was built to predict COVID-19 infection compared to controls, with AUC taken as performance metric. The model was built using the replication cohort (AIID), it was not tested on Discovery (AIID) or Arden cohorts as they lacked non-infected patient information. As shown in Fig. 3, a Gaussian Naïve Bayes model distinguishing controls vs. COVID-19 patients utilising 10-fold cross-validation, achieves an excellent prediction performance (0.91 AUC). The model is composed of Peaks 22, 33, 56 and 25. With VIF values all below 3 indicating there is no issue of collinearity. No covariates were included in the model, as age and sex were not deemed to influence the peaks included.

### The *N*-glycome is reflective of ICU admission risk

**Fig. 4:**
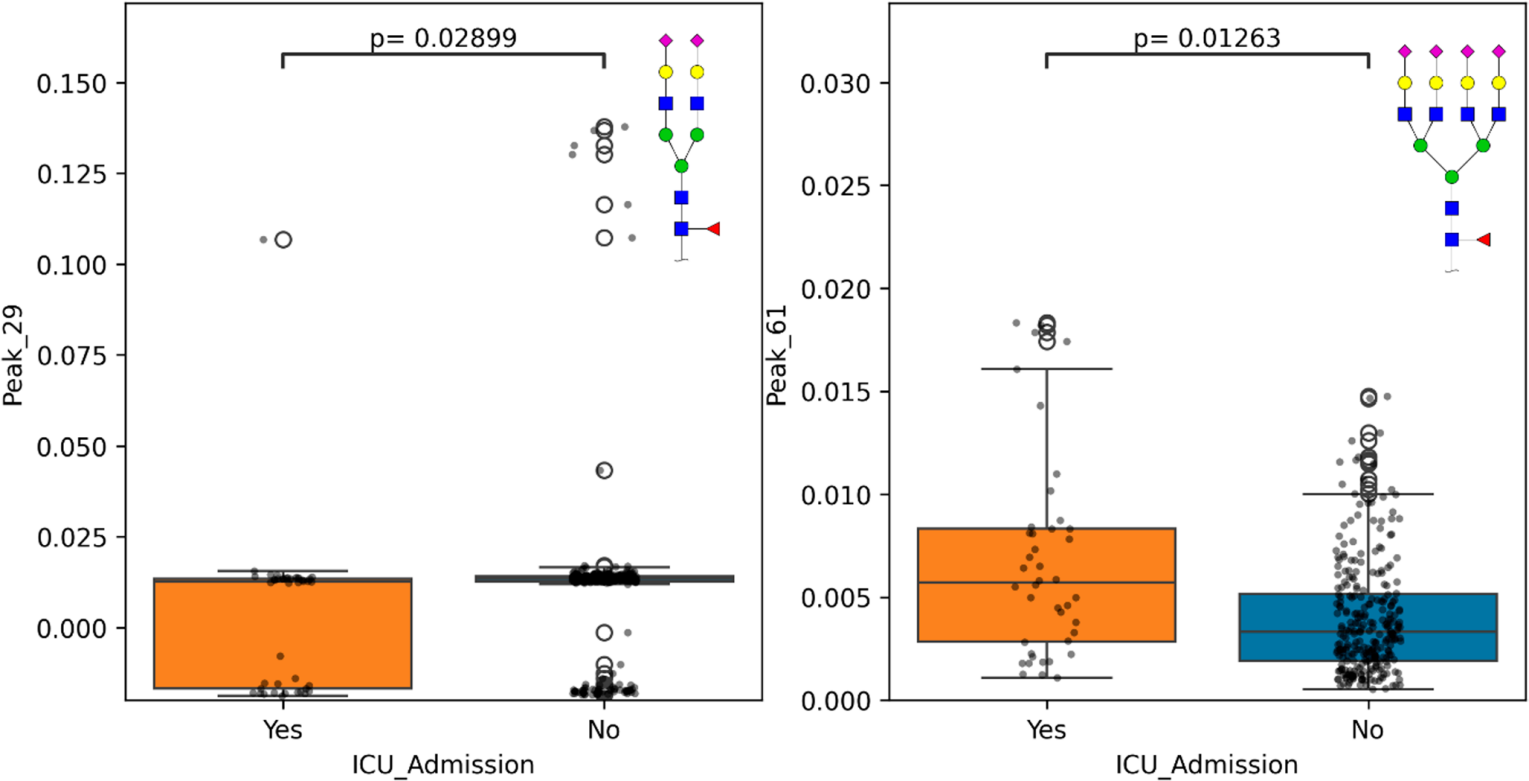
Boxplots depicting the glycan peaks (y-axis relative area) significantly different across ICU admission (x-axis) with selection criteria (|fold-change| > 1.5 and *p*-value< 0.05 after Benjamini-Hochberg Correction). Glycan structures represented

Glycan peaks (62) extracted from plasma *N*-glycan analysis were tested for significant difference across ICU admission over the course of COVID-19 disease (Document S4 Tables 7, 8 and 9). The discovery cohort offered 236 individuals (no admission = 212, ICU admission = 24), which was validated with the replication cohort offering 87 patients (no admission = 63, ICU admission = 24). A total of 5 glycan peaks (Peak 26/A2G2S2 (*p*-value = 0.013), 29/A2G2S2F (*p*-value = 0.09), 35/A3G3S2 (*p*-value = 0.013), 37/A3G3S2 (*p*-value = 0.013), 61/A4G4S4F (*p*-value = 0.013)) were found to be statistically significant (*p*-value < 0.05) after Benjamini-Hochberg Correction (Figure S7). Of these 5 glycan peaks, the biantennary and triantennary glycans were elevated in those who were admitted to the ICU, whilst the tetrantennary glycans were lowered. Further selection criteria were applied (|fold-change| > 1.5) and narrowed peaks of interest to Peak 29 and Peak 61, as shown in Fig. 4. CRP, is used as a baseline comparison of an inflammatory marker; however, it failed to be significant across ICU admission (*p*-value = 0.286) (Figure S7).

**Fig. 5:**
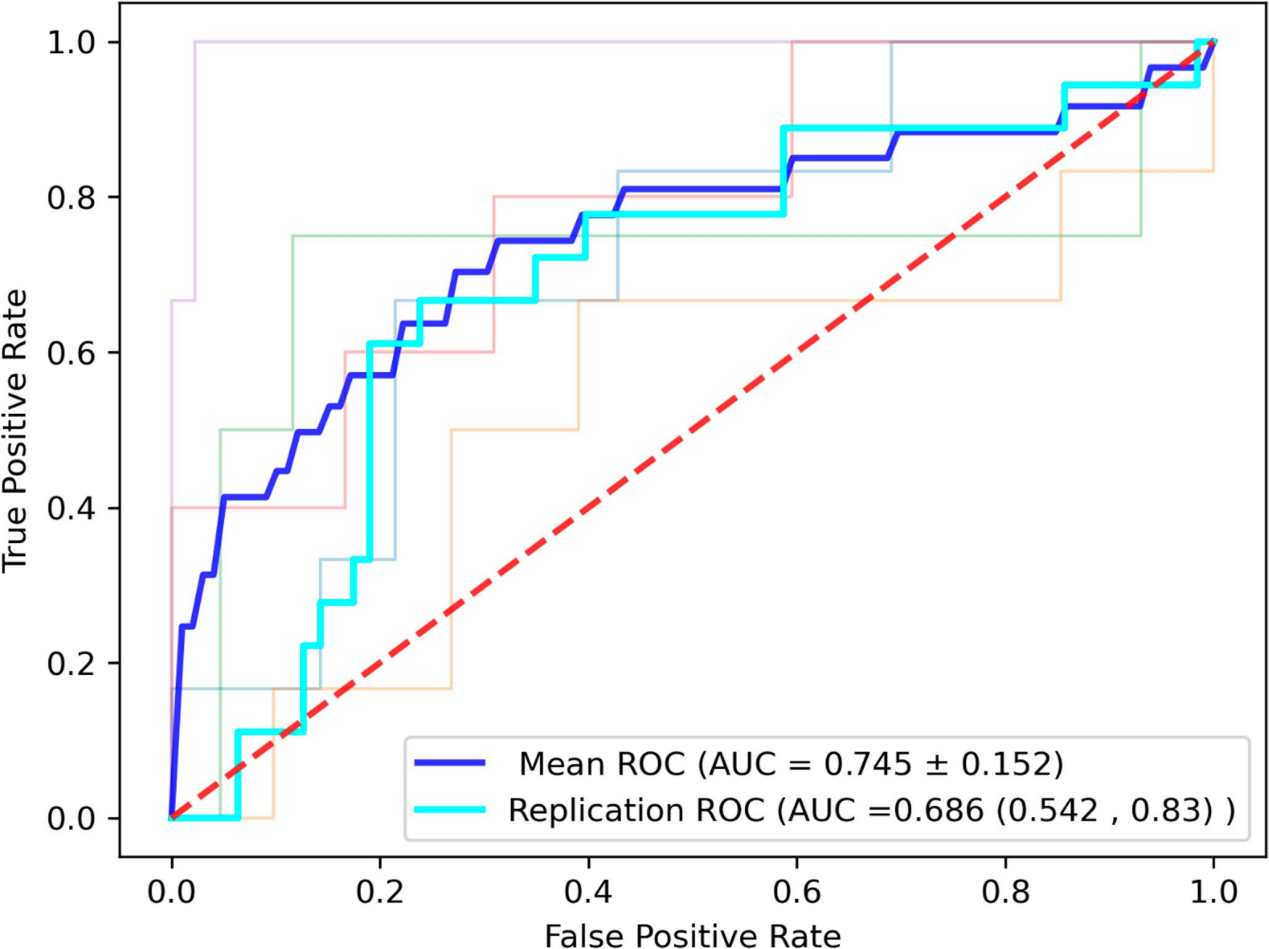
A ROC curve demonstrating the predictive power of an ICU Gaussian NB with sample weights classification model. The model is built on a combination of clinical and glycan markers (Peak 26, 58 and 59 and IL-18). Model was trained on discovery cohort, producing the mean ROC AUC based on the average performance of the 5-fold cross-validation and tested on replication cohort, producing replication ROC AUC. The dashed red line represents an AUC of 0.5. All other coloured lines are the performance of each fold in the cross-validation

A multivariate model predicting ICU admission during COVID-19 disease course was built through sequential feature selection, considering both glycomic and clinical variables, training on discovery cohort (AIID) and validating on the replication cohort (AIID), as shown in Fig. 5. The Arden biobank was not used in this model, as it does not contain the clinical marker, IL-18, needed. ICU admission can be predicted moderately well with a gaussian naïve bayes classifier with sample weight inclusion to compensate for unbalanced proportions of ICU admitted patients (discovery cohort 0.75 AUC, replication cohort 0.69 AUC) with a combination of clinical and glycan markers (Peak 26, 58,59 and IL-18). VIF was employed for collinearity checks, with the highest value being 1.11 and so not a point of concern. No covariates were included. Although performance is moderate, it boasts an improvement on an IL-18 model, with 0.55 AUC under 10-fold cross-validation (Document S4 Table 8).

### *N*-glycans signatures distinguish COVID-19 mortality

**Fig. 6:**
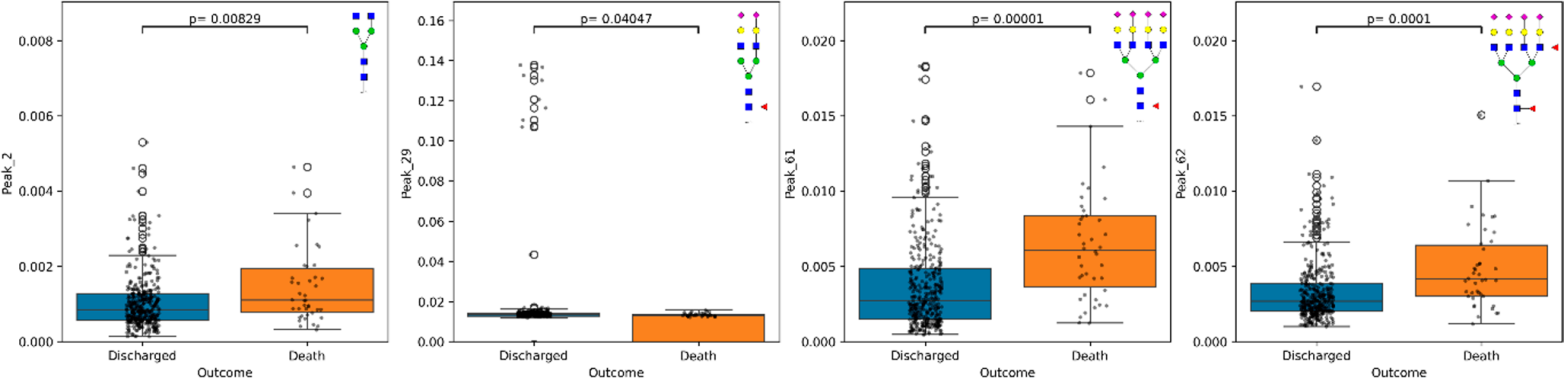
Glycans significantly different across mortality, with selection criteria (|fold-change| > 1.5 and *p*-value< 0.05 after Benjamini-Hochberg Correction. *P*-value from a Mann-Witney U test comparing patients who died and patients who were discharged glycan peaks, with adjustment for multiple correction. Mortality outcome on the x-axis and relative area of glycan on the y-axis

To further understand which glycans are associated with COVID-19 severity, the UHPLC-FLD-detected 62 *N*-glycans of patients who were discharged were compared to those who died (Document S4 Tables 10, 11 and 12). The discovery cohort offered 259 individuals (discharged = 228, death = 31), which was validated with the replication cohort offering 90 individuals (discharged = 79, death = 11). 23 direct trait glycans significantly differ in those who died compared to those that did not, these include Peak 61 (*p*-value 0.000007, 0.800 AUC) and Peak 17 (A2G2F) (*p*-value 0.00005, 0.769 AUC). Malignancy was more frequent in mortality (12% in discharged, 45% in death) and statistically different (*p*-value 0.000003, 0.664 AUC). Interestingly, clinical markers such as CD40 (*p*-value 0.00002, 0.785 AUC) and P-Selectin (*p*-value 0.00005, 0.788 AUC) were decreased in those who died. Further selection criteria (|fold-change| > 1.5 and *p*-value < 0.05 after Benjamini-Hochberg Correction) was applied, revealing 4 glycan peaks to be strongly distinguished by COVID-19 disease outcome, as shown by Fig. 6.

Consistent with ICU admission, glycan Peak 29 (FA2G2S2) was decreased in patients who died compared to those discharged. Alike to COVID-19 disease against controls and ICU admission, Peak 61 (FA4G4S4) was increased in patients experiencing a more severe disease course. In addition, glycan Peaks 2 (A2) and 62 (A4G4S4F) were raised in patients who died. Glycomic biomarkers proved to be more sensitive at distinguishing hospital outcome compared to the baseline clinical marker, CRP (*p*-value = 0.33759), which failed to be significant (figure S8).

**Fig. 7:**
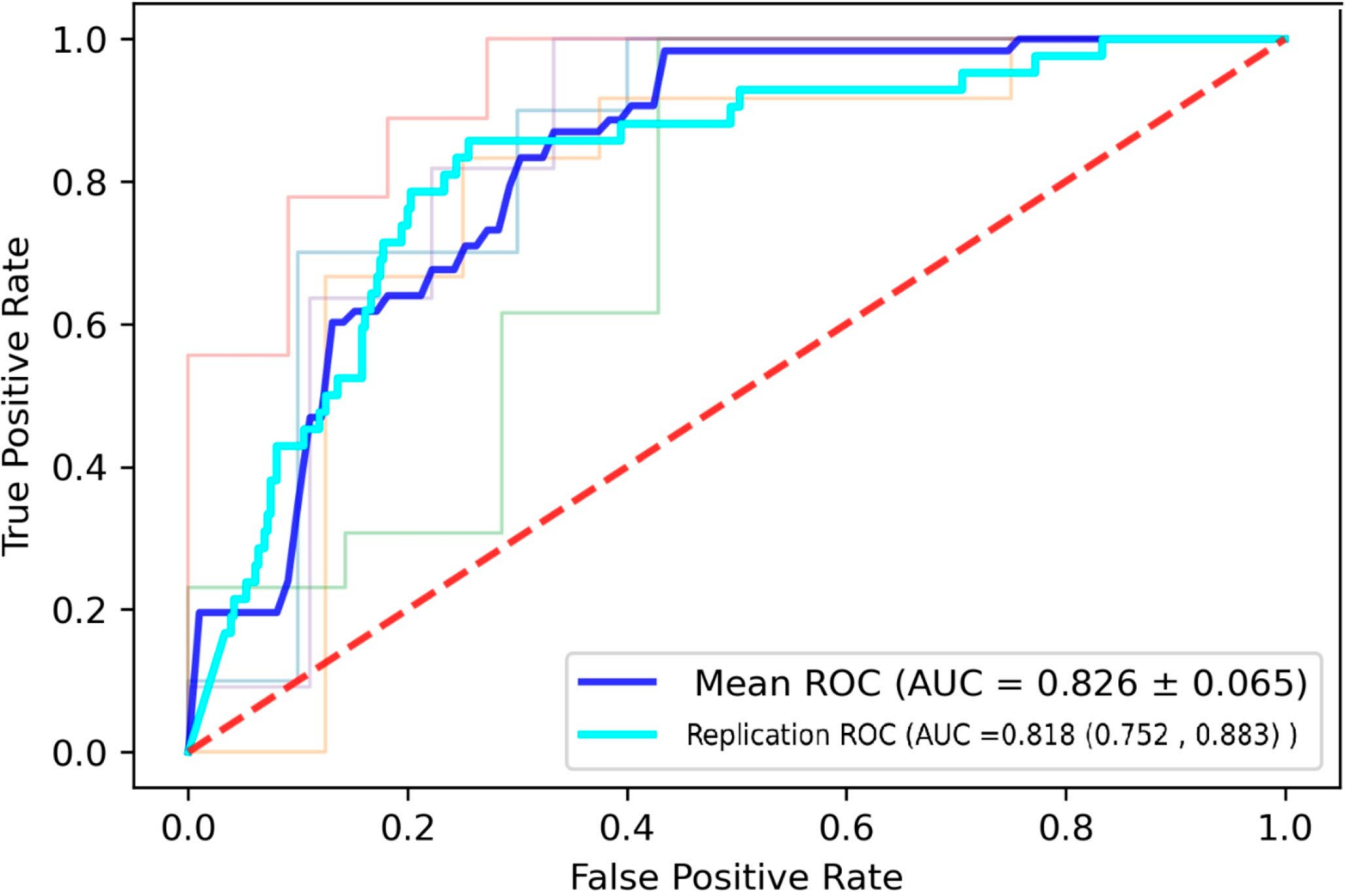
A ROC curve displaying Gaussian Naïve Bayes classifier with immunosuppressed, Age, Peak 31 and 61 predicting mortality. The model was trained on the Arden biobank, the average of the 5-fold cross validation is represented as the blue line, Mean ROC. The model was tested on the combined discovery and replication cohort, the performance represented as the light blue line labelled Replication ROC. A 5-fold cross-validation with AUC as performance metric. The dashed red line represents an AUC of 0.5. All other coloured lines are the performance of each fold in the cross-validation

A multivariate model predicting disease outcome (death/discharge) was built using metadata and glycomic markers through sequential feature selection. The model was trained on the Arden Biobank cohort, due to its balanced nature (45 discharged, 55 died), tested via a combination of discovery (AIID) and replication cohort (AIID). A combination of glycans, Peak 31, 61 and immunosuppressed (No/Yes) and Age, predicted mortality well (Arden biobank 0.83 AUC, discovery and replication cohort 0.82 AUC) with a Gaussian Naïve Bayes classifier, as shown in Fig. 7. Sex was not included as a covariate as the male/female ratio is balanced across groups (discharged: 164 F,196 M, death: 18 F, 24 M). The immunosuppressed variable considers whether patients have a immunosuppressive condition, including HIV, recorded as no or yes. Using discovery (AIID) and replication (AIID) cohort to train and test the model respectively, followed by validation on the Arden Biobank cohort, was also performed (Figure S9) with a good performance across cohorts seen (Discovery 0.826 AUC, Replication 0.703 AUC).

## Discussion

We have demonstrated that HILIC quantification of plasma *N*-glycans can portray the presence of SARS-CoV-2 infection and in turn, implies the disease course of patients. Developments to HILIC have progressed *N*-glycan analysis, allowing 62 glycan peaks to be detected and imply the inflammatory status of individuals. This study’s strength lies in the replication of biomarkers, with considerably sized cohorts (discovery cohort 236, replication cohort 87, Arden Biobank 100 samples) establishing the glycosylation trends seen in the course of COVID-19 disease are consistent and potentially representative of complex biological pathways involved.

A study by Bladergroen et al., composed of 169 COVID-19 patients and 12 healthy controls produced a total plasma *N*-glycosylation profile from mass spectrometry [27]. Validating glycans eluded from this study will not only prove the reproducibility of COVID-19 biomarkers but the robustness of glycans eluted by different quantification machinery. Bladergroen et al. has validated the majority of glycans (8 of the 11) found to distinguish controls from infected patients in our study. For example, one of their strongest predictors was A4G4S4 (H7N6E3L1) [27], which was also selected by our criteria, as A4G4S4 resides under the significant Peaks 56 and 57. The majority of these glycans were replicated in a previous study, with concordant trends across case vs. control aside from Peaks 9 (A2BG1, 0.761 AUC, adj *p* = 0.00342) and 23 (A2BG2S1,0.786 AUC, adj *p* = 0.0007), which we found to be decreased in COVID-19 patients, whilst reported to be elevated in the previous study [27]. In addition, Peak 25 was deemed significant in our study, identified as H5N5S1F1/FA2BG2S1 through MS glycan assignment, but it was not highlighted as a strong candidate in a previous study [27]. These discrepancies may be due to the controls being hospitalised patients, whilst Bladergroen et al., study used individuals which were not hospitalised. In addition, the balancing of a cohort can also impact results. Of the significant glycans, those which are bisected were decreased in COVID-19 patients compared to controls, whilst fucosylated triantennary glycans and tetra-antennary glycans were elevated. These glycomic trends were also consistent with a Bladergroen et al. [27]. A multivariate model of glycan peaks allowed for a strong prediction of COVID-19 disease compared to controls (0.907AUC). This distinct *N*-glycan profile in SARS-CoV-2 infected individuals establishes plasma *N*-glycan composition is reactive to infection and well represents the inflammatory changes compared to hospitalised patients without COVID-19, which can be further categorised by comparing the prognosis of individuals. However, the controls represent individuals who have been hospitalised and so their glycomic profile is not equitable to non-hospitalised controls; yet do ensure glycomic alterations seen in SARS-CoV-2 infected is specific to the infection than opposed to more general inflammatory markers which are also experienced by non-infected hospitalised patients.

There has been extensive research into how IgG glycosylation is associated with COVID-19 and its severity. Inspection of IgG glycosylation directly relates to the humoral immune response and is clearly interpreted as glycans presented on the Fc domain of IgG, which can alter interactions with a plethora of immune cells. For example, increased agalactosylation of IgG regulates the proinflammatory characteristics of IgG through activation of the complement system and has been found to increase in cases of severe COVID-19[16]. Elevated levels of inflammatory glycans, such as sialic acid and Lewis oligosaccharide [38], increase over time in cases of hospitalised COVID-19 patients and correlated to receptor binding domain antibody levels [18]. In addition to IgG, many acute phase proteins involved in the immune response against SARS-CoV-2 are glycoproteins[39–41]. To broaden our understanding of how glycoproteins and the presentation of specific glycan structures represent the biological pathways involved in viral infection, plasma *N*-glycans must be analysed. The *N*-glycan composition of acute phase proteins present in sera are malleable to inflammation. Very distinct *N*-glycosylation alterations are witnessed in high branching (tri and tetra-antennary) glycans on haptoglobin, A1AGP, alpha-1-antitrypsin and alpha-1-antichymotrypsin in states of inflammation[42–44]. This upregulation of high branching glycans is mediated by an influx of hexosamine biosynthetic pathway, which drives UDP-GlcNAc (the substrate involved in high branch construction) in hepatocytes [45] and increases high branching of acute phase proteins [46]. A proposal of protein associations with glycans can be made through assessing previously reported work; however these are speculations and would require further studies to confirm. Comparing TPNG of hospitalised patients with or without SARS-CoV-2 infection revealed the diagnostic marker, FA3G2S1 (Peak 25, 0.822 AUC) to be lowered in COVID-19 patients. FA3G2S1 is present on immunoglobulin E (13.5%) and immunoglobulin M (26%)[29]. IgG and IgM production during SARS-CoV-2 typically becomes detectable within 2 weeks of symptom onset, therefore within the context of this study, antibody levels may be a predeterminant of disease severity [47]. FA3G2S1 has appeared as disease biomarker in previous studies, such as Parkinson’s disease in males [48].

Further comparison of SARS-CoV2 infected individuals with those who are not, highlighted glycan Peaks 41, identified as A3G3S3, Peaks 56,57,59 contain A4G4S4, 60 and Peak 61 are FA4G4S4, to be higher in COVID-19 patients. All of which are potentially present on the glycoprotein A1AGP, as inferred from previous studies [29] and perhaps by association, A1AGP protein is raised in COVID-19 patients. In a previous study, A1AGP proved to be raised in those with COVID-19 compared to healthy controls, in addition to those who were admitted to the ICU and with a mortality outcome [49]. A1AGP is an acute-phase protein indicative of systemic injury and inflammation, capable of impeding neutrophil migration[49, 50]. As part of the innate immune response, neutrophils are one of the first responses to infection, producing NETs which minimise pathogen mobility [51]. Concordant with our study, A1AGP glycoprotein levels were shown elevated in severe COVID-19 and in vitro studies, reduced NETosis and upregulated IL-6[49]. This is thought to be linked to the cytokine storm seen in COVID-19[52]. Glycan Peak 41 could also be present on the glycoprotein, haptoglobin [29]. Serum haptoglobin has been shown to be decreased in critical cases of COVID-19[53], potentially because of hemolytic anemias, with hemolysis initiated by SARS-CoV-2 infection of erythrocytes releasing hemoglobins. Other studies have proposed haptoglobin levels positively correlate with COVID-19 severity, however failed to show statistical significance [30]. In addition to A1AGP, Peaks 60 and 61 (FA4G4S4) can also reside on ceruloplasmin. Ceruloplasmin can act as an acute phase protein, being raised in inflammatory environments and has been shown to correlate with COVID-19 severity [54]. The ability of *N*-glycans to predict disease course and raise awareness of patients likely to escalate to severer symptoms, resulting in ICU admission or death, would provide the opportunity for earlier intervention and preparation. Analysis of the prognostic value of *N*-glycans in terms of COVID-19 disease course revealed a lowered level of FA2G2S2 (Peak 29) for both ICU admission (0.600 AUC) and mortality (0.777 AUC). FA2G2S2 also appears in other disease biomarker studies, such as, in clear cell renal cell carcinoma, where FA2G2S2 on the immunoglobulin J chain, has a positive correlation with increasing tumour stages [55]. In addition, IgG FA2G2S2 has been explored in correlation to age, showing a negative correlation with both females and males [56]. It is evident FA2G2S2 has a relationship with inflammation, this is further evidenced by its involvement in response to immunosuppressant therapy such as a lower level of IgG FA2G2S2 in sera of patients after vedolizumab treatment compared to untreated presented in unpublished work [57].

FA2G2S2 is present on alpha-1-antritripsin (3.6%), alpha-2-macrogobulin (15%), ceruloplasmin (14.5%), hemopexin (5%), serotransferrin (2.5%), vitronectin (3.3%), immunoglobulin D (7.6%), immunoglobulin G (16%) immunoglobulin E (25%)[29]. Whilst IgE accommodates the highest composition of FA2G2S2, it may not be relevant in this case as the clearance of terminal *N*-glycan sialic acids, such as FA2G2S2, on IgE depletes its functionality in effector cell degranulation and so has an anti-inflammatory effect [58]and thus a positive relationship between FA2G2S2 and COVID-19 severity would be expected[59, 60]. Interestingly, a deficiency in alpha-1-antitripsin (AAT), of which FA2G2S2 is present [29], is associated with COVID-19 severity [61], hospitalisation [62] and mortality [63]. Hospitalised patients with severe COVID-19 had reduced plasma levels than those admitted for non-COVID-19 pneumonia [64], which is concurrent with our results as Peak 29/FA2G2S2 (adj *p* = 0.09) was significantly lower in COVID-19 patients compared to non-COVID-19 patients (controls). AAT is a serine protease inhibitor, thought to inhibit, the transmembrane protease, TMPRSS2, required for SARS-CoV-2 cell entry [65, 66]. Thus, we could postulate that FA2G2S2 is present on AAT and the depleted levels we detect across COVID-19 infected vs. controls, in those admitted to ICU and those who die are due to a deficiency in AAT.

A4G4S4, although not distinguished in ICU admitted patients, was significant when comparing hospitalised individuals with or without SARS-CoV-2 infection (Peaks 56 and 57). Similarly, Bladergroen et al., study has found isomers of A4G4S4, H7N6E4 (adj p 2.2e-06) and general tetra-antennary sialylation, TA4S (*p*-value = 2.4e-7), to be significantly different across ICU admission and controls [27]. It has been demonstrated that the diagnostic glycan trend, namely raised levels of FA4G4S4 (Peak 61, 0.796 AUC), was also observed in those admitted to the ICU (0.711 AUC) and who died (0.800 AUC). FA4G4S4 has only been shown to be present on A1AGP across the APPs [29]. In cases of severe disease and compared to healthy controls, levels of AAG have been elevated[30, 49]. AAG seems to represent inflammatory pathways as levels correlate with IL-6 and CRP, specifically NETosis and IL-6 expression, potentially as part of the cytokine storm seen in severe COVID-19[49]. Thus, the elevation of A4G4S4 could represent the raise in AAG seen in severe COVID-19 disease.

A combination of glycans and metadata performed very well at predicting mortality in COVID-19 patients (Arden Biobank cohort 0.83 AUC, discovery and replication cohort 0.82 AUC). The advantage of training classification models on a balanced cohort (Arden Biobank) was seen when comparing training the model on the discovery cohort (0.83 AUC), experiencing a drop in performance during validation by the replication cohort (0.70 AUC).

TPNG shows promise in unveiling diagnostic and prognostic biomarkers for COVID-19 disease, reinforced through the application of internal (Replication) and external (Arden Biobank) cohorts used to replicate glycomic patterns seen. Whilst SARS-CoV-2 incidences and the associated mortalities have dramatically reduced, this work serves as an example of the uses of TPNG in the context of viral disease infection and the consequential disease course. This work adds to the evidence of TPNG as a useful tool for biomarkers of diseases, as shown in diabetes [19], systemic lupus erythematosus [20], colorectal cancer [21], cardiovascular disease [22] and inflammatory bowel disease [23]. Focussing on early strain and first infection samples offers a simple interpretation of the data, uncomplicated by the further infections and vaccinations incurred over the pandemic, which may affect the glycomic profile. These results offer a record of the impact of first infection on the TPNG, which could later be compared to the TPNG of patients infected by different strains, quantifying the evolution of the disease. This study of TPNG widens the search of biomarkers from the well characterised IgG glycome and aims to pull attention to the value of TPNG analysis in the context of disease, which is reinforced, with glycans outcompeting standard inflammatory markers, such as CRP. TPNG analysis has the advantage of avoiding additional purification steps compared to the analysis of isolated glycoproteins in addition, the integration and quantification of UHPLC-FLD data is less taxing than UHPLC-FLD-MS processing. Further work is needed to understand the biological pathways the glycan signals are partaking in, specifically the glycoproteins they are presented on. In addition, it would be interesting to determine the consistency of the TPNG signatures in patients further infected with the current strains of SARS-CoV-2 and, for those experiencing it, examine the impact of long covid on their glycosylation.

## Limitations of the study

TPNG of patient samples prior to infection were not analysed and so the differential signals driven by COVID-19 cannot be concluded. In addition, it has not been established whether these signals are COVID-19 specific or would apply to other infections. A comparative study of the glycomic profile of patients with other infections would be interesting. ICU admission is arguably a subjective decision, depending on the hospital protocol, and so the clinical application of ICU prediction models would be less translatable than mortality outcome. Employing the use of several cohorts to train and test models, provides an indication of the reproducibility of the classifiers. However, there were limitations in the cohorts. Namely, the unbalanced nature of the number of patients who were admitted to the ICU and experienced mortality in the discovery and replication cohort. This issue was compensated by using sample weights and in the case of the mortality model, training the classifier on a more balanced cohort, the Arden biobank cohort. Although TPNG offers a broader insight into the biological mechanisms at play, without glycoproteomics following this analysis, there can be no assurance the glycomic patterns witnessed are due to specific speculated mechanisms. To confirm these associations in future work, a feasible strategy would be to selectively enrich the candidate proteins from serum. Commercial antibodies against both A1AGP and ceruloplasmin are available and can be immobilised onto either a protein G support or a cyanogen bromide–activated Sepharose matrix. Plasma from individuals representing the strongest and weakest glycan signatures attributed to these proteins would be applied to the matrices for immuno-affinity enrichment. Released glycans from the captured proteins would then be profiled using our standard analytical workflow, and statistical association modelling performed to directly test the proposed links.

## Supplementary Information

Below is the link to the electronic supplementary material.

Supplementary file 1 (PNG 51.7 KB)Figure S1: Bar graph depicting glycan peaks (x-axis) and retention time (y-axis) of the average retention times of the standards for each cohort, represented by colours purple for discovery, red for replication and green for Arden Biobank. Where peaks experienced splitting, they are represented as a and b

Supplementary file 2 (PNG 27.3 KB) Figure S2: A bar graph depicting glycan peaks (y-axis) and relative area (x-axis) of the average relative area of the standards run for each cohort, represented by colours purple for discovery, red for replication and green for Arden Biobank. Where peaks experienced splitting, they are represented as a and b

Supplementary file 3 (PNG 69.7 KB) Figure S3: An example of a chromatogram of procainamide-labelled plasma N-glycans. The X-axis presenting retention time, whilst Y-axis presents relative area (%). Document S2, expands on glycan assignment for each peak, providing glycan nomenclature, glycan structure and selection criteria

Supplementary file 4 (PNG 98.1 KB) Figure S4: PCA depicting the cohort variation across discovery (orange), replication (blue), controls (green) and the Arden Biobank (red). Each point represents a patients’ relative area of all 62 peaks.

Supplementary file 5 (PNG 92.9 KB) Figure S5: A scatterplot depicting 2 PCA components based on the relative abundance of the 62 glycan peaks for each patient in the discovery and replication cohort. Datapoints (patients) coloured purple are controls, blue are those who were admitted to ICU, whilst red is not. Diamond symbol represents patients who were discharged, whilst a cross represents death.

Supplementary file 6 (PNG 91.6 KB) Figure S6, A boxplot of CRP (y-axis) across COVID-19 disease infection (x-axis) is utilised as a baseline predictor. *P*-values have been produced from Mann Witney U test with multiple testing correction

Supplementary file 7 (PNG 374 KB) Figure S7, Boxplots depicting glycans which are significantly different across ICU admission after multiple testing correction (*p*-value< 0.05) prior to further selection criteria (|fold change| > 1.5). In addition, a boxplot of CRP across ICU admission is utilised as a baseline predictor

Supplementary file 8 (PNG 31.9 KB) Figure S8: A boxplot of CRP (y-axis) across hospitality outcome (x-axis) is utilised as a baseline predictor. *P*-values has been produced from Mann Witney U test with multiple testing correction. **Multivariate Models for Mortality Prediction**Both glycomic and clinical data was entered into feature selection, the model with the highest performance and replication was selected. A logistic regression model with features Peak 58, age and S100B was selected. Although training performance on discovery cohort was good (0.845 AUC), consistency was weaker than a glycan only based model, with validating on replication cohort dropping to 0.703 AUC. The Arden Biobank could not be used in this model due to unavailable clinical data

Supplementary file 9 (PNG 105 KB) Figure S9: A ROC curve depicting a mortality prediction classifier, based on glycomic and clinical data. Model was trained on discovery cohort with 5-fold cross-validation (Mean ROC) and validated on the replication cohort (Replication ROC), taking AUC as performance metric. Red dashed line represents 0.50 AUC. Thin lines represent performance of each fold from cross-validation. *N*-Glycan variation across COVID-19 severity. Glycan peaks (62) extracted from *N*-glycan analysis were tested for significant difference across COVID-19 disease severity (Document S4 Table 3-6), whilst considering the natural order of mild to critical disease. The discovery cohort offered 310 individuals (mild=151, moderate=66, severe=42, critical=51), which was validated with the replication cohort offering 92 individuals (mild=40, moderate=22, severe=15, critical=15). A total of 35 direct traits were found to be statistically significant after Benjamini-Hochberg Correction. These glycans were then further tested for the predictive power of prognostics.The greatest univariate predictors of WHO severity were Peak 61 with mild 0.647 AUC, moderate 0.533 AUC, severe 0.622 AUC and 0.704 AUC for critical with a 5-fold 10 repeat cross-validation. In addition, Peak 59 (*p*-value 0.0000, mild 0.664 AUC, moderate 0.552 AUC, severe 0.652 AUC, critical 0.667 AUC) and 57 (*p*-value 0.000, mild 0.695 AUC, moderate 0.602 AUC, severe 0.627 AUC, critical 0.658 AUC) were strongly significant

Supplementary file 10 (PNG 150 KB) Figure S10: Boxplots of the glycan peaks with the greatest univariate predictors of WHO severity (Mild<moderate<severe<critical), with adjusted *p*-values and glycan structures represented. A boxplot depicting CRP across COVID-19 severity is also provided.**Prognostic capabilities of the Glycome**The discovery cohort was used to train a classification model predicting WHO severity, which was then validated through the replication cohort. The feature selection methods, such as recursive feature elimination, strive to achieve the highest AUC seen in both discovery and replication. A 10-fold cross-validation was employed for the training phase of the model and a weighted average provided across the one-vs-rest models for each disease severity subgroup.A Linear Discriminant Analysis built on Peaks 40, 55, 61 and 62 model performed moderately well, compared to CRP (0.587 AUC)

Supplementary file 11 (PNG 187 KB) Figure S11: (A) A ROC curve depicting predictive performance of WHO severity based on discovery cohort, with one-vs-rest approach to gaining a predictive performance (AUC) for each disease severity, using a 10-fold cross-validation

Supplementary file 12 (PNG 171 KB) (B) A ROC curve depicting predictive performance of WHO severity based on replication cohort, with one-vs-rest approach to gaining a predictive performance (AUC) for each disease severity

Supplementary file 13 (XLSX 63.8 KB)

Supplementary file 14 (XLSX 751 KB)

Supplementary file 15 (XLSX 29.8 KB) Document S3: Tables 1,2,3 and 4 denoting the patient summary of metadata, clinical markers and glycomics for the Discovery, Replication, Arden Biobank and Controls, respectively

Supplementary file 16 (XLSX 74.8 KB) Document S4. Tables 1,2 and 3 display the univariate analysis for controls vs COVID-19 patients, for glycomics, metadata and clinical markers, respectively. Tables 4,5, and 6 display the univariate analysis for the glycomics, clinical markers and metadata respectively, across WHO severity groups (mild, moderate, severe, critical). Tables 7,8,9 consider the univariate analysis across ICU admission and not, for glycomics, clinical and metadata, respectively. Tables 10,11 and 12 consider univariate analysis across mortality for glycomics, metadata and clinical markers, respectively. Table 13 compares glycan peaks significance across control compared to infected, comparing severity groups, ICU compared to non-ICU admitted and death compared to discharged

## Data Availability

No datasets were generated or analysed during the current study.
