# Supplementary figures and images for: Total plasma N-glycomic patterns of COVID-19 disease
Source: Glycoconj J. 2026 Jan 20;43(1):9. doi: 10.1007/s10719-025-10201-1 (PMC12819545; doi:10.1007/s10719-025-10201-1)

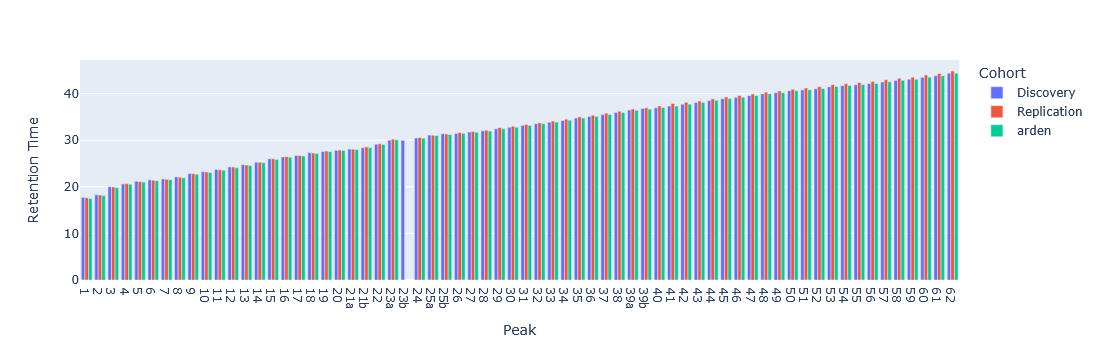

Supplement: Supplementary file 1 — (PNG 51.7 KB)Figure S1: Bar graph depicting glycan peaks (x-axis) and retention time (y-axis) of the average retention times of the standards for each cohort, represented by colours purple for discovery, red for replication and green for Arden Biobank. Where peaks experienced splitting, they are represented as a and b [file 10719_2025_10201_MOESM1_ESM.png]

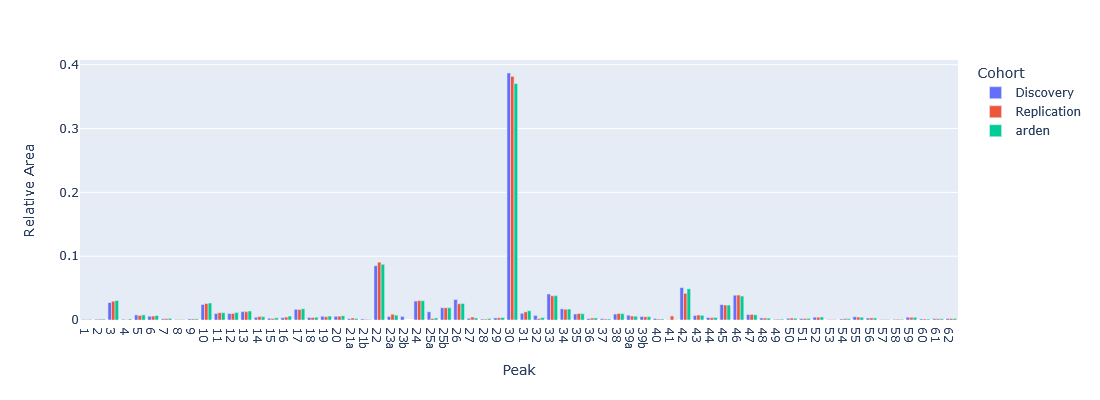

Supplement: Supplementary file 2 — (PNG 27.3 KB) Figure S2: A bar graph depicting glycan peaks (y-axis) and relative area (x-axis) of the average relative area of the standards run for each cohort, represented by colours purple for discovery, red for replication and green for Arden Biobank. Where peaks experienced splitting, they are represented as a and b [file 10719_2025_10201_MOESM2_ESM.png]

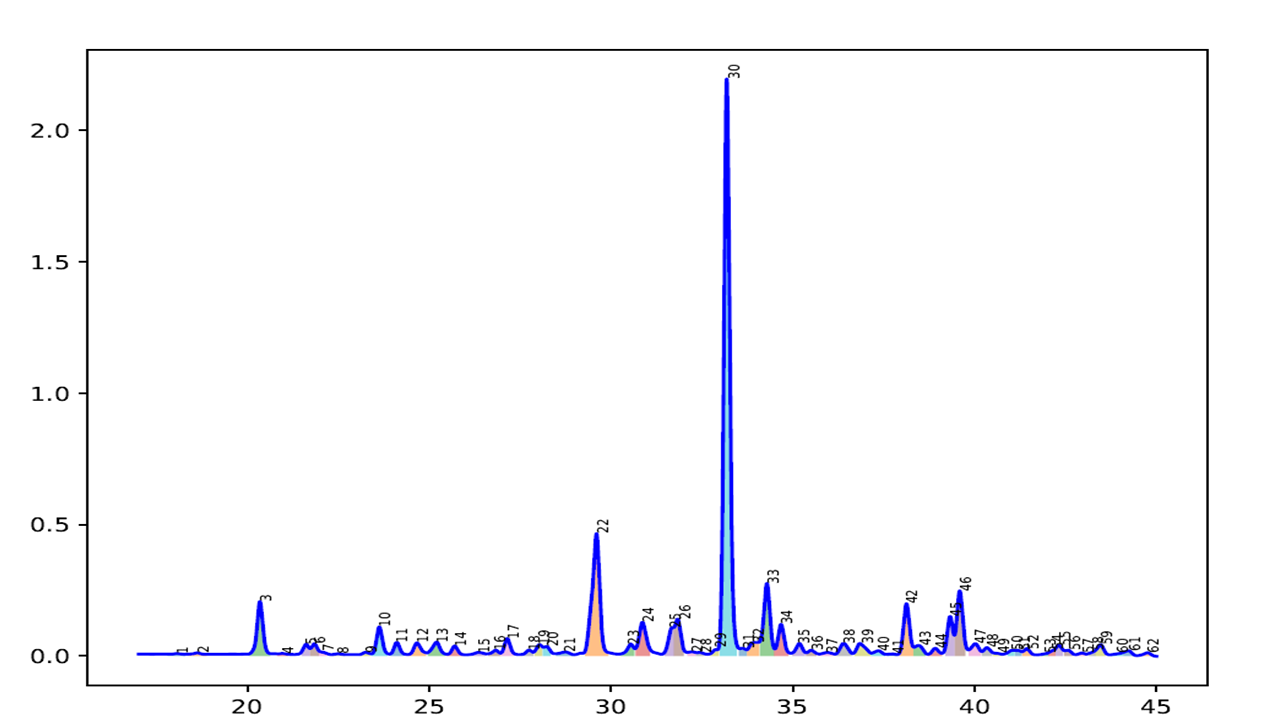

Supplement: Supplementary file 3 — (PNG 69.7 KB) Figure S3: An example of a chromatogram of procainamide-labelled plasma N-glycans. The X-axis presenting retention time, whilst Y-axis presents relative area (%). Document S2, expands on glycan assignment for each peak, providing glycan nomenclature, glycan structure and selection criteria [file 10719_2025_10201_MOESM3_ESM.png]

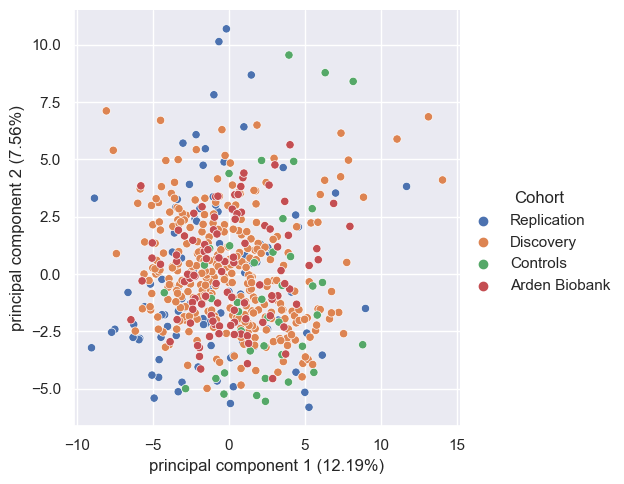

Supplement: Supplementary file 4 — (PNG 98.1 KB) Figure S4: PCA depicting the cohort variation across discovery (orange), replication (blue), controls (green) and the Arden Biobank (red). Each point represents a patients’ relative area of all 62 peaks. [file 10719_2025_10201_MOESM4_ESM.png]

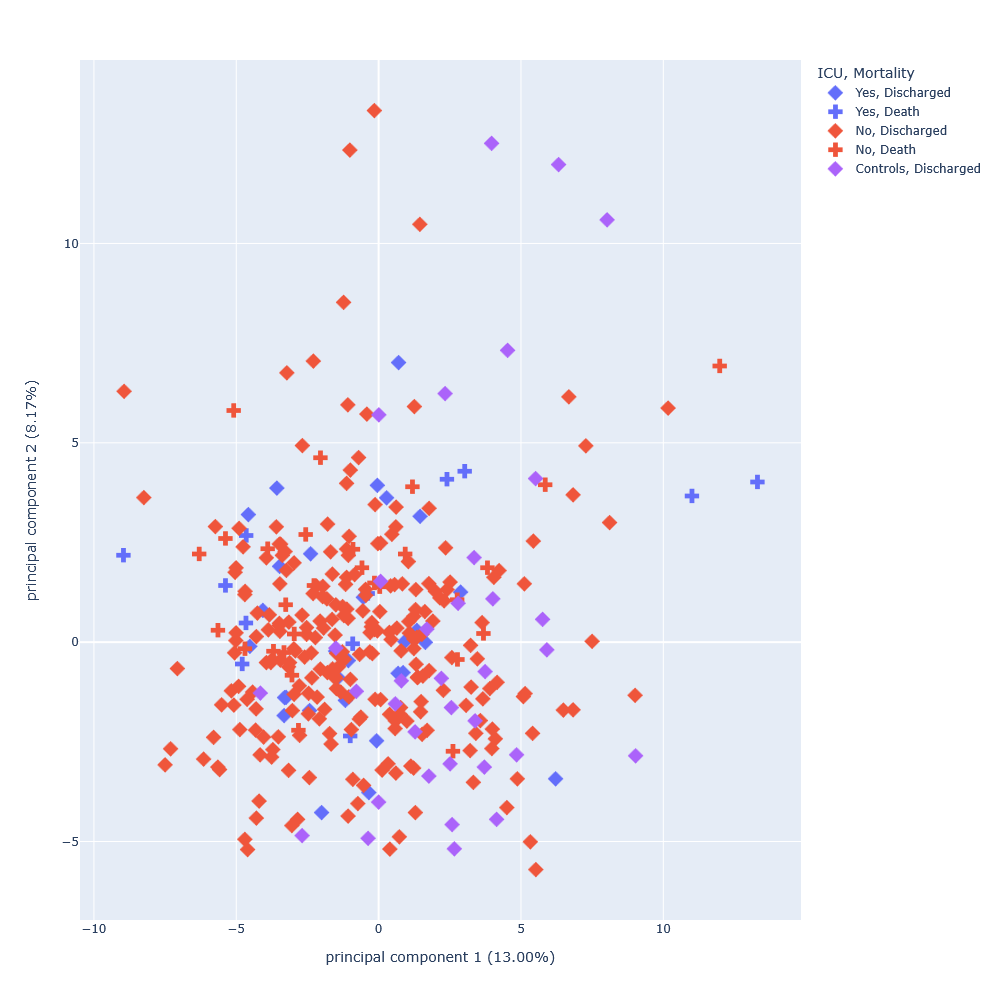

Supplement: Supplementary file 5 — (PNG 92.9 KB) Figure S5: A scatterplot depicting 2 PCA components based on the relative abundance of the 62 glycan peaks for each patient in the discovery and replication cohort. Datapoints (patients) coloured purple are controls, blue are those who were admitted to ICU, whilst red is not. Diamond symbol represents patients who were discharged, whilst a cross represents death. [file 10719_2025_10201_MOESM5_ESM.png]

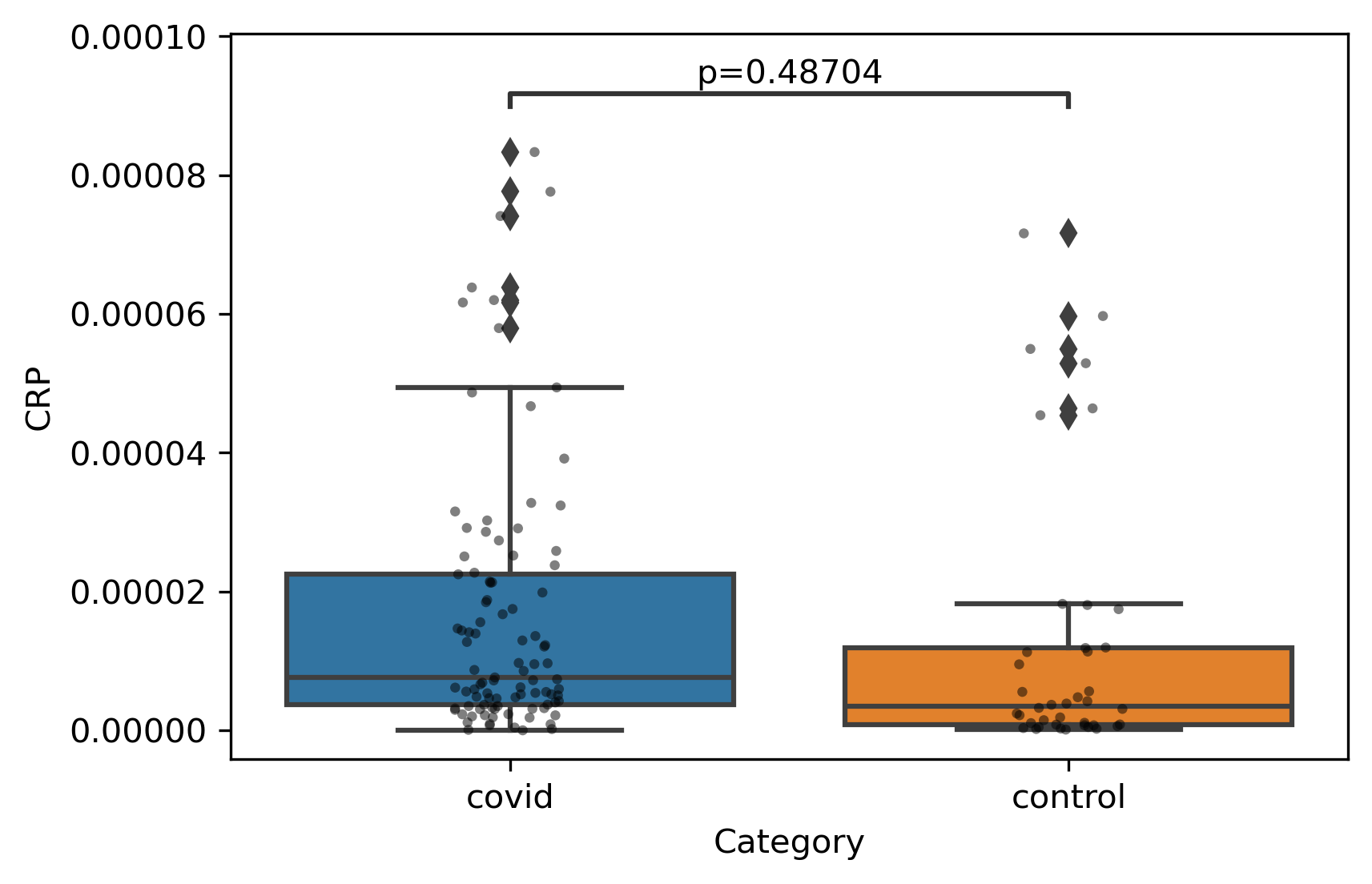

Supplement: Supplementary file 6 — (PNG 91.6 KB) Figure S6, A boxplot of CRP (y-axis) across COVID-19 disease infection (x-axis) is utilised as a baseline predictor. P-values have been produced from Mann Witney U test with multiple testing correction [file 10719_2025_10201_MOESM6_ESM.png]

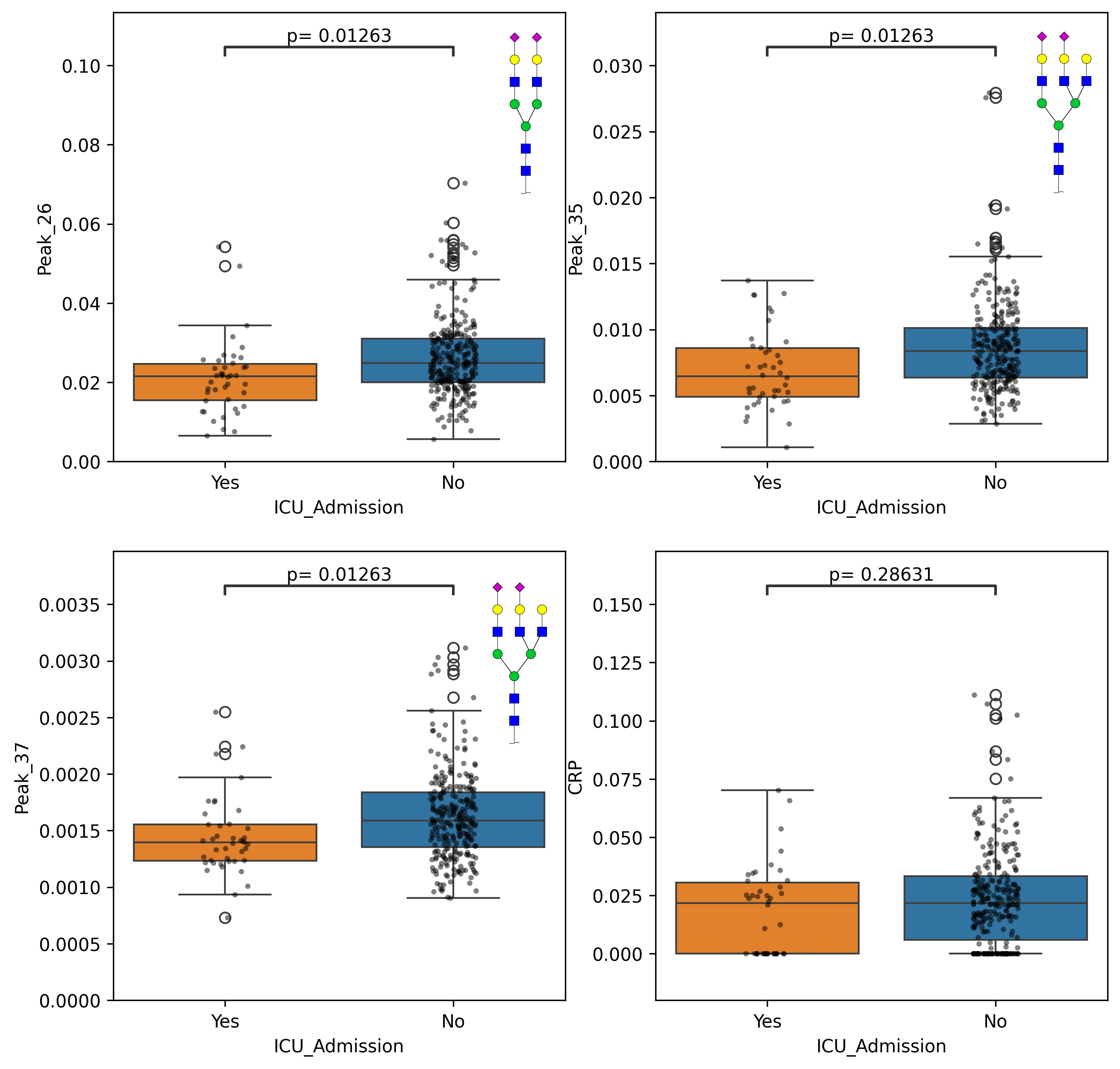

Supplement: Supplementary file 7 — (PNG 374 KB) Figure S7, Boxplots depicting glycans which are significantly different across ICU admission after multiple testing correction (p-value< 0.05) prior to further selection criteria (|fold change| > 1.5). In addition, a boxplot of CRP across ICU admission is utilised as a baseline predictor [file 10719_2025_10201_MOESM7_ESM.png]

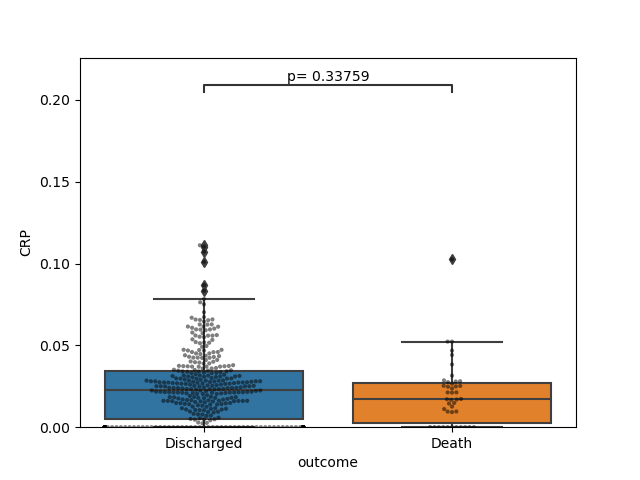

Supplement: Supplementary file 8 — (PNG 31.9 KB) Figure S8: A boxplot of CRP (y-axis) across hospitality outcome (x-axis) is utilised as a baseline predictor. P-values has been produced from Mann Witney U test with multiple testing correction. Multivariate Models for Mortality PredictionBoth glycomic and clinical data was entered into feature selection, the model with the highest performance and replication was selected. A logistic regression model with features Peak 58, age and S100B was selected. Although training performance on discovery cohort was good (0.845 AUC), consistency was weaker than a glycan only based model, with validating on replication cohort dropping to 0.703 AUC. The Arden Biobank could not be used in this model due to unavailable clinical data [file 10719_2025_10201_MOESM8_ESM.png]

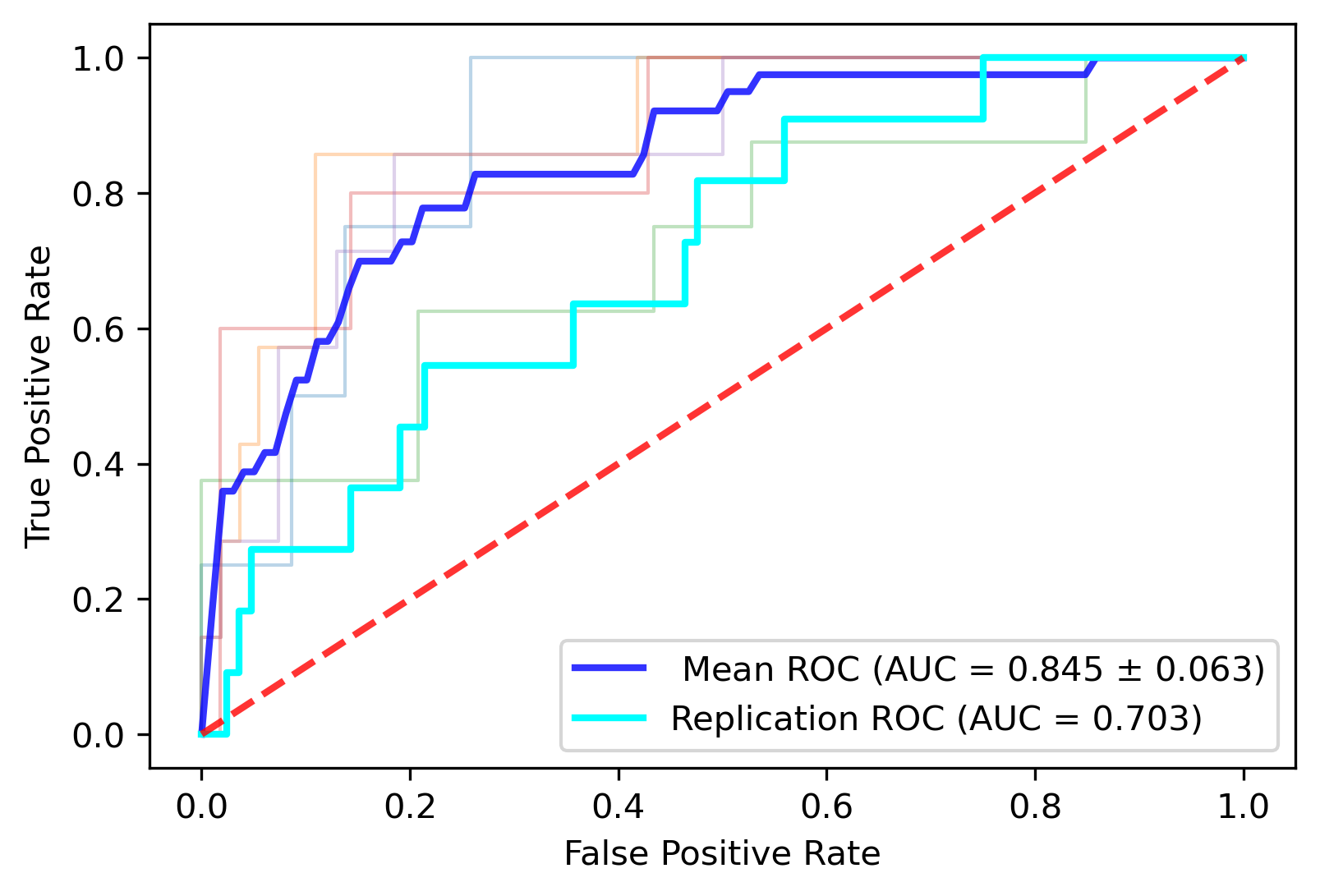

Supplement: Supplementary file 9 — (PNG 105 KB) Figure S9: A ROC curve depicting a mortality prediction classifier, based on glycomic and clinical data. Model was trained on discovery cohort with 5-fold cross-validation (Mean ROC) and validated on the replication cohort (Replication ROC), taking AUC as performance metric. Red dashed line represents 0.50 AUC. Thin lines represent performance of each fold from cross-validation. N-Glycan variation across COVID-19 severity. Glycan peaks (62) extracted from N-glycan analysis were tested for significant difference across COVID-19 disease severity (Document S4 Table 3-6), whilst considering the natural order of mild to critical disease. The discovery cohort offered 310 individuals (mild=151, moderate=66, severe=42, critical=51), which was validated with the replication cohort offering 92 individuals (mild=40, moderate=22, severe=15, critical=15). A total of 35 direct traits were found to be statistically significant after Benjamini-Hochberg Correction. These glycans were then further tested for the predictive power of prognostics.The greatest univariate predictors of WHO severity were Peak 61 with mild 0.647 AUC, moderate 0.533 AUC, severe 0.622 AUC and 0.704 AUC for critical with a 5-fold 10 repeat cross-validation. In addition, Peak 59 (p-value 0.0000, mild 0.664 AUC, moderate 0.552 AUC, severe 0.652 AUC, critical 0.667 AUC) and 57 (p-value 0.000, mild 0.695 AUC, moderate 0.602 AUC, severe 0.627 AUC, critical 0.658 AUC) were strongly significant [file 10719_2025_10201_MOESM9_ESM.png]

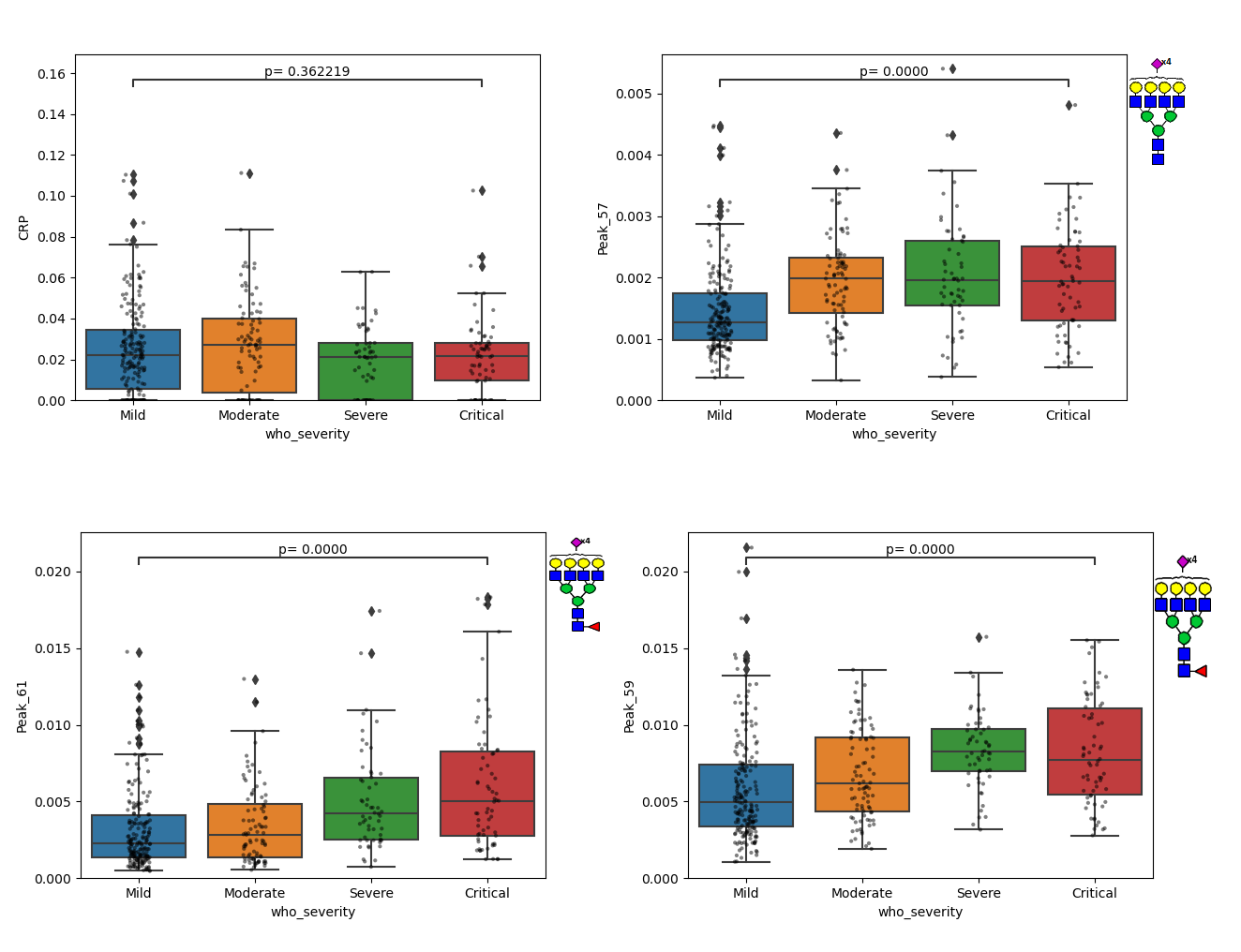

Supplement: Supplementary file 10 — (PNG 150 KB) Figure S10: Boxplots of the glycan peaks with the greatest univariate predictors of WHO severity (Mild<moderate<severe<critical), with adjusted p-values and glycan structures represented. A boxplot depicting CRP across COVID-19 severity is also provided.Prognostic capabilities of the GlycomeThe discovery cohort was used to train a classification model predicting WHO severity, which was then validated through the replication cohort. The feature selection methods, such as recursive feature elimination, strive to achieve the highest AUC seen in both discovery and replication. A 10-fold cross-validation was employed for the training phase of the model and a weighted average provided across the one-vs-rest models for each disease severity subgroup.A Linear Discriminant Analysis built on Peaks 40, 55, 61 and 62 model performed moderately well, compared to CRP (0.587 AUC) [file 10719_2025_10201_MOESM10_ESM.png]

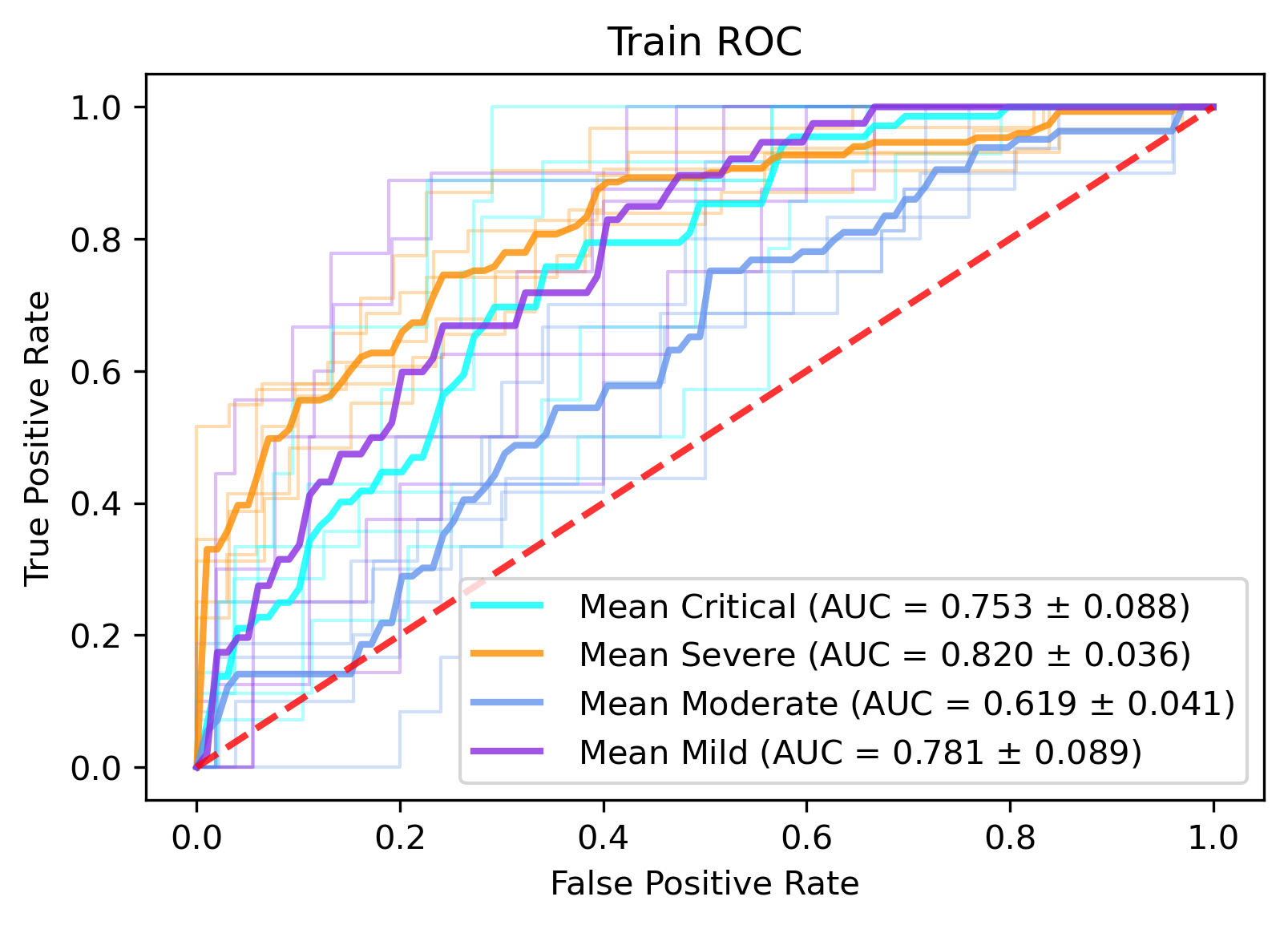

Supplement: Supplementary file 11 — (PNG 187 KB) Figure S11: (A) A ROC curve depicting predictive performance of WHO severity based on discovery cohort, with one-vs-rest approach to gaining a predictive performance (AUC) for each disease severity, using a 10-fold cross-validation [file 10719_2025_10201_MOESM11_ESM.png]

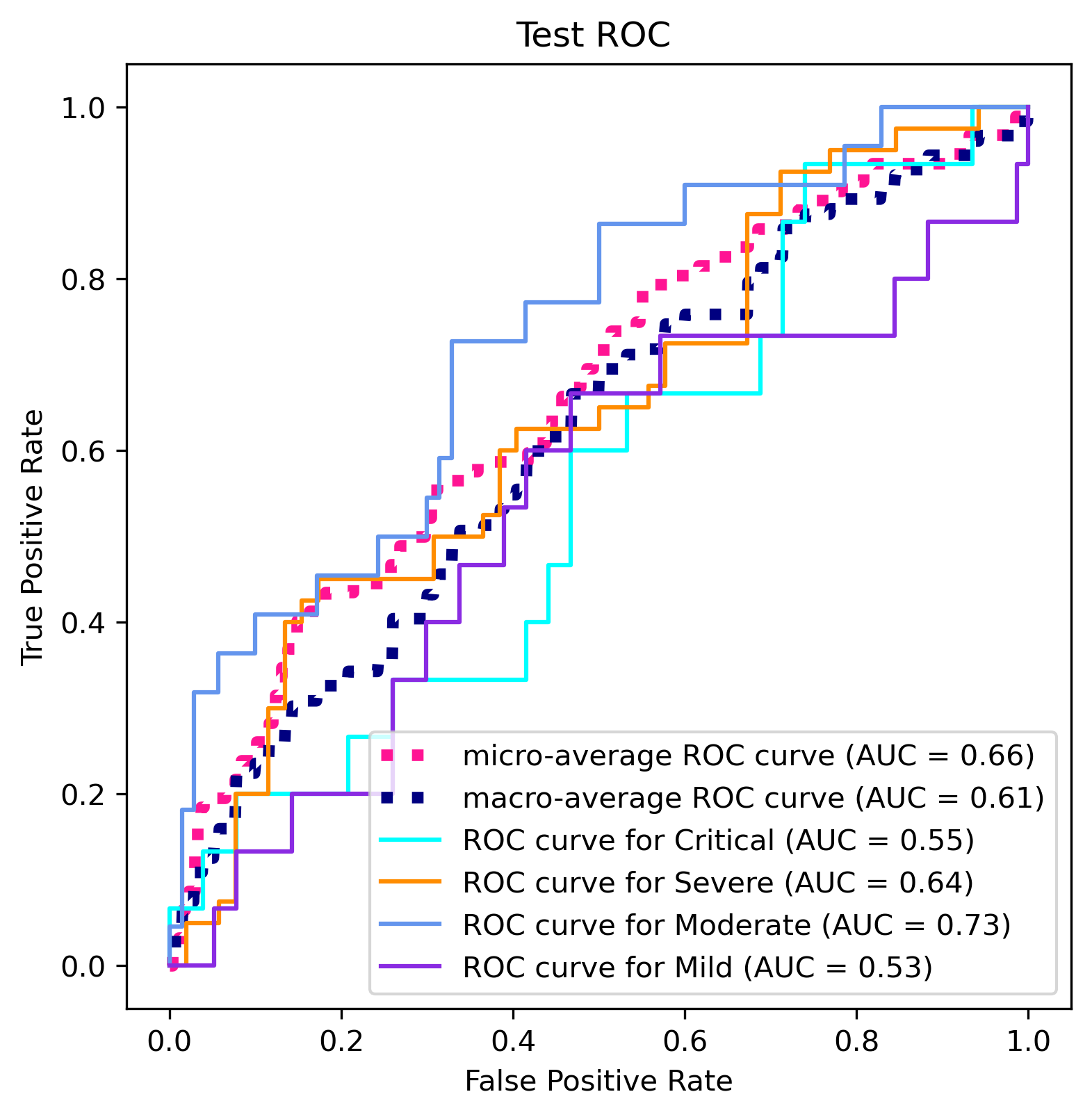

Supplement: Supplementary file 12 — (PNG 171 KB) (B) A ROC curve depicting predictive performance of WHO severity based on replication cohort, with one-vs-rest approach to gaining a predictive performance (AUC) for each disease severity [file 10719_2025_10201_MOESM12_ESM.png]
